# Morphometric‐Assisted Prediction of Developmental Toxicity Using Stem Cell‐Based Embryo Models in Microwells

**DOI:** 10.1002/adhm.202404847

**Published:** 2025-05-09

**Authors:** Vinidhra Shankar, Athanasia Zoi Pappa, Clemens van Blitterswijk, Erik Vrij, Stefan Giselbrecht

**Affiliations:** ^1^ MERLN Institute for Technology‐Inspired Regenerative Medicine Department of Cell Biology‐Inspired Tissue Engineering (cBITE) Maastricht University Maastricht 6229ET The Netherlands; ^2^ Department of Obstetrics and Gynaecology Maastricht University Medical Centre Maastricht 6229ET The Netherlands; ^3^ GROW Research Institute for Oncology and Reproduction Maastricht University Maastricht 6229ET The Netherlands

**Keywords:** developmental and reproductive toxicity (DART), high throughput screening, microwells, stem cell‐based embryo models, teratogens

## Abstract

Congenital abnormalities cause ≈3% of fetal defects and premature deaths in Europe, often due to maternal exposure to toxicants. To mitigate the ethical and logistical challenges of animal studies, stem cell‐based models are being exploredthat offer scalable readouts at various stages of embryogenesis. However, most current in vitro models are limited in complexity, throughput, automation compatibility or real‐time spatio‐temporal read‐outs. In this study, a scalable, automated platform capable of imaging and quantifying morphological features such as shape, size, texture, and marker intensity is presented. Using a microwell screening platform, XEn/EpiCs, a peri‐implantation stage embryo model that mimics eXtraembryonic Endoderm and Epiblast co‐development, is robustly generated and used to screen a library of 38 reported compounds. Unlike conventional cytotoxicity assays, this approach also evaluates development‐disrupting morphological changes, termed “morphotoxicity”, thereby offering complementary insights that may improve the prediction of developmental toxicity across cell types. This pilot study shows thathigh doses of compoundslike retinoic acid, caffeine, ampyrone, and dexamethasone, significantly disrupt XEn/EpiC development, causing morphotoxic effects with or without affecting cell viability. Together, thisstudy highlights the importance of complementing cytotoxicity assessments with morphotoxicity read‐outs, emphasizing its potential to enhance the evaluation of teratogenic risks in toxicity tests.

## Graphical abstract

This proof‐of‐concept study involves high‐throughput teratogenicity screening of compounds using XEn/EpiCs, a 3D stem cell‐based embryo model, within microwells. The term ‘morphotoxicity’ is introduced to complement traditional cytotoxicity assays through automated feature extraction and machine‐learning‐assisted classification of morphologies. The authors’ findings reveal novel compound‐specific morphological effects, demonstrating faster, more accessible, and improved imaging‐based evaluations of toxic compounds in the future.

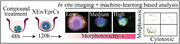

## 1 Introduction

Developmental and reproductive toxicity (DART) refers to the induction of physical or functional defects in an embryo or fetus, affecting their development or causing infertility due to exposure to teratogens.^[^ ^1^ ^]^ Teratogens can be synthetic or natural chemicals and biologics, which upon maternal exposure, cause serious malformations in the embryo or fetus.^[^ ^2^ ^]^ It is usually manifested by developmental delay, structural abnormalities, or lethality caused by direct ingestion or indirect exposure to toxic compounds through the environment.^[^ ^3^ ^]^ Most commonly known teratogens include alcohol, recreational drugs, and medications, as well as endocrine disruptors, such as bisphenol‐A (BPA), found in most plastics.^[^ ^4^ ^]^ It is important to note that any substance can be teratogenic depending on the concentration, time, and duration of exposure of the toxic substance to the developing embryo.^[^ ^5^ ^]^ The understanding of the implications of chemicals passing through the placental barrier and reaching the embryo or fetus has fueled the research toward an improved understanding of their effect on overall reproductive and developmental health.^[^ ^6^ ^]^

Pharmaceutical drugs intended for use in pregnant women must be screened for teratogenicity before market release to ensure the safety of the developing fetus. Until recently, these screenings have been performed in animal models, most commonly in rodent (rat and mouse) models, for which generally thousands of embryos are sacrificed to clinically approve a drug, emphasizing the need for alternative models to predict adverse effects. Recently, the promotion of the use of alternatives to animal models for toxicity testing and the ethical use of animals based on the 3Rs—Replace, Refine, and Reduce in DART assessment, has put an added focus on the micro‐physiological systems providing potential strategies to achieve this.^[^ ^7^ ^]^

Candidate teratogens are studied in animals for their potential to cause developmental abnormalities. However, wide, unbiased screening is prohibited due to the large numbers of animals required. The introduction of a new model for DART assessment requires a thorough validation of the specific effects of compounds, focusing on the time, dose, and duration of exposure that induce toxicity, as introduced by Daston and colleagues.^[^ ^8^ ^]^

Stem cell‐based embryo models have emerged as a powerful tool to study early mammalian embryogenesis.^[^ ^9^ ^]^ Their ability to be generated in a scalable and reproducible manner positions them as an effective strategy to be used for high‐throughput DART testing. These models, generated from a pluripotent population of cells in a controlled microenvironment, possess the ability to recapitulate some features of natural embryonic development robustly. Capitalizing on these properties, there have been different types of stem cell‐based embryo models used in DART screening that focus on different stages and features of the embryo model in mouse and human cells.^[^ ^10^ ^]^ The work on mouse stem cell‐based embryo models is extremely valuable to compare and validate the in vitro data with the studies on rodents to streamline the potential applications of these models.^[^ ^11^ ^]^

A vast number of read‐outs in DART assays in vitro commonly focus on some of the indicators of developmental toxicity by assessing cell death and cytotoxicity using calorimetric, fluorometric, or luminescence assays. There have also been other kinds of read‐outs based on the cell‐specific differentiation or proliferation effects upon exposure to specific toxicants.^[^ ^12^ ^]^ However, embryonic development is more compounded, and simpler 2D in vitro assays do not account for all potential risks for multicellular morphogenetic effects. The limitation is in the integrated analysis of tissues, which is currently done in animal models, thereby putting more emphasis on the in vitro models to also provide information on the effect of compounds on morphogenesis and spatiotemporal organization of different tissues. By leveraging stem cell‐based embryo models, we are at the brink of a new era where such readouts become feasible not only in in vivo but also in in vitro models. However, often more complex 3D in vitro cultures are limited in their throughput, in turn, limiting their translation to screening in the clinical domain. Such studies would require a scalable, reproducible model with a multi‐tissue/multi‐organ level of complexity that can enable both cellular and morphological read‐outs to test a large library of compounds. Here, we address both issues through our new platform that can extract morphological information from images of the 3D in vitro model in a high‐throughput manner.

Machine learning‐based phenotyping of embryo model systems can help elucidate naturally occurring variance and induced changes in the developmental progression of embryos.^[^ ^13^ ^]^ Importantly, automated phenotyping of multi‐tissue development permits increasing experimental throughput and thereby enables classifying molecular and genetic drivers by their phenotypic defects.^[^ ^14^ ^]^ We recently showed the formation of a 3D in vitro stem cell‐based embryo model that recapitulates epiblast (Epi) and extraembryonic endoderm (XEn) co‐development, named XEn/EpiCs, mimicking a peri‐implantation stage embryo^[^ ^15^ ^]^ that can be generated in a high‐throughput microwell set‐up,^[^ ^16^ ^]^ facilitate in situ imaging, and automate statistical analysis. Here, we assess the impact of a custom teratogenic library composed of known and potential teratogens (Table S1, Supporting Information) on the morphogenesis of XEn/EpiCs at different concentrations. We start by screening for teratogenic effects on the embryo model using three concentrations of compounds, referencing the available sources from the literature to evaluate the range of toxicity that different compounds exert during development.

Morphology‐based quantification of features, or morphometrics, provides valuable insights into how external perturbations affect the integrated and coordinated growth of different tissue types. This approach is particularly important for predicting developmental toxicity as it offers a more nuanced understanding of teratogenic effects beyond standard cytotoxicity measures. In this study, we utilize a morphology‐based approach to identify the compounds that induce a significant morphological change to the formation of XEn/EpiCs and affect its developmental progression, an effect we term as morphotoxicity. We propose that complementing traditional cytotoxicity assays with morphotoxicity read‐outs presents a more comprehensive strategy for toxicogenomics and developmental and reproductive toxicity (DART) assessments.

## 2 Results

### 2.1 Designing the Library of Known and Potential Teratogens

To create our screening compound library, we started with compounds that have been classified by the Food and Drug Administration (FDA) as causing a teratogenic effect on the development of an embryo/fetus. Compounds or drugs are stratified into five distinct FDA Categories, namely A, B, C, D, and X,^[^ ^17^ ^]^ reflecting a gradient of toxicity determined through validation tests in model organisms, which ranges from minimal or absent effects (Category A) to severe malformations or lethality (Category X) (**Table** **1**). The final library chosen in this study includes 28 compounds (Table S1, Supporting Information) in different categories of teratogenicity, such as vitamins (ascorbic acid and retinoic acid), antihistamines (propafenone hydrochloride), antibiotics (penicillin and isoniazid), NSAIDs (ibuprofen), pesticides/disinfectants (2,4,6‐triiodophenol and carbamazepine), chemotherapeutic drugs (5‐fluorouracil and busulfan), and other known teratogens to humans (thalidomide).

**Table 1:** FDA's examination of pregnancy labeling. The regulations require that each product be classified under one of five pregnancy categories (A, B, C, D, or X) based on the risk of reproductive and developmental adverse effects or, for certain categories, based on such risk weighed against potential benefit.^[^ ^17b^ ^]^

FDA pregnancy categories	Toxicity levels	Studies conducted
A	No known risk to the fetus during all trimesters	Studies in pregnant women and animal reproduction studies
B	No known risk to the fetus; however, no adequate and well‐controlled experiments in pregnant women have been conducted; should be used during pregnancy only if clearly needed.	Animal reproduction studies
C	Adverse effects on the fetus; however, no adequate and well‐controlled experiments in pregnant women have been conducted; should be used during pregnancy only if the potential benefit justifies the potential risk to the fetus.	Animal reproduction studies
D	Positive evidence of human fetal risk based on adverse reaction data from investigational or marketing experience or studies in humans, but the potential benefits from the use of the drug in pregnant women may be acceptable despite its potential risks.	Animal reproduction studies and human studies
X	Demonstrated fetal abnormalities or there is positive evidence of fetal risk based on adverse reaction data from investigational or marketing experience or studies in humans; the risk of the use of the drug in a pregnant woman clearly outweighs any possible benefit	Animal reproduction studies and human studies

The concentrations used in the screen were chosen based on literature findings on the effect of teratogens on mouse embryonic development, as well as the concentrations used for testing with in vitro embryo models. For the compounds that had no reference concentration tested in mouse embryos or in vitro models, the known data from other model organisms in volume per kg were calculated to estimate an approximate range for the screen.

The screening was performed in two levels, of which the first step involved the identification of the concentration range at which the compound causes an effect on the development of XEn/EpiCs. The second step in screening involved expanding the range to five concentrations to find the minimum and maximum dosages of compounds to impart a significant reduction in the formation of XEn/EpiCs using the automated morphology detection. Each compound in this pilot experiment was tested across three independent replicates.

### 2.2 Screening of Three Concentrations of Teratogens to Assess the Level of Developmental Toxicity on XEn/EpiCs

**Figure 1:**
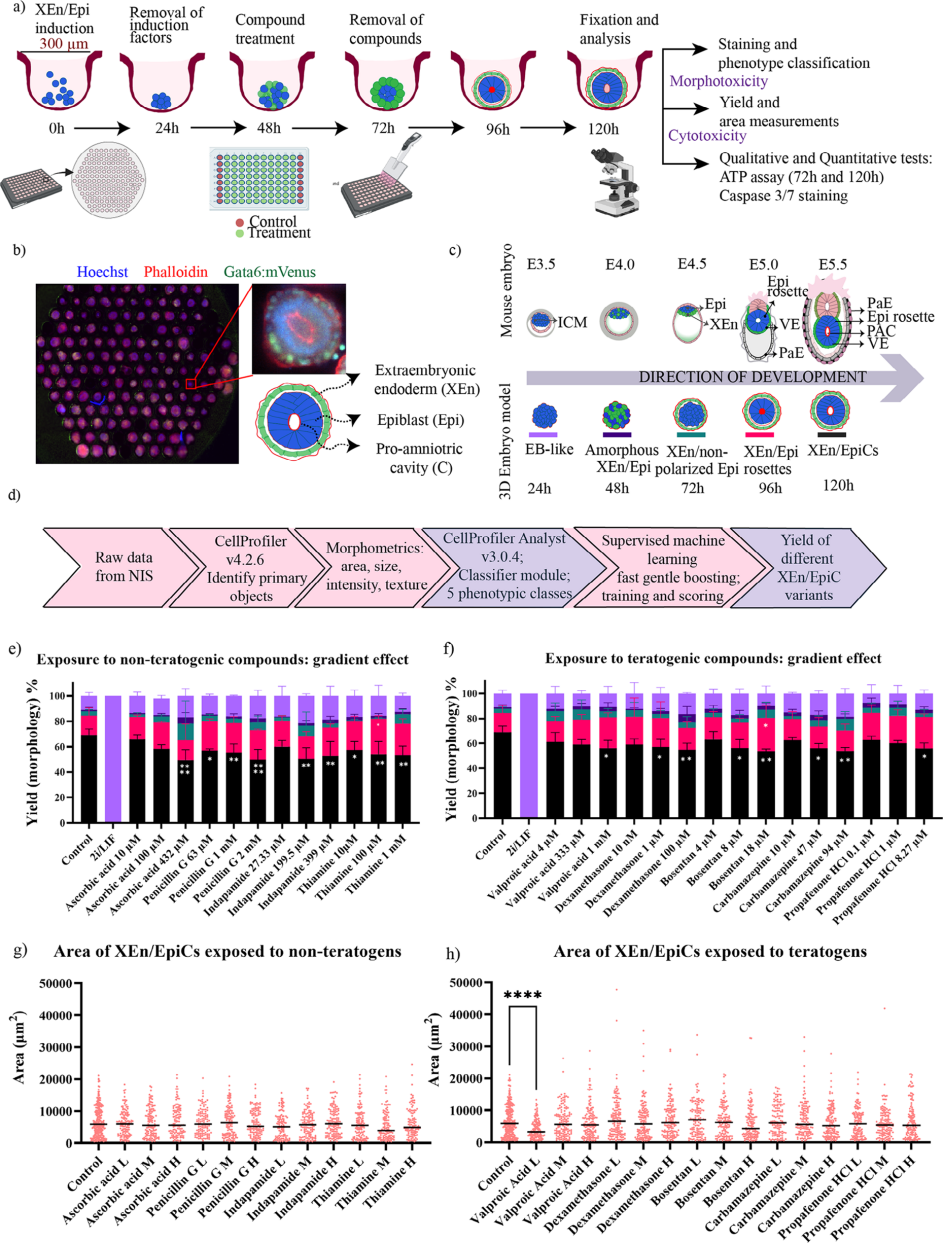
Screening of three concentrations of teratogens to assess the level of developmental toxicity on XEn/EpiCs: a) schematic of the protocol for the formation of XEn/EpiCs within microwells, time of addition of teratogens, and the read‐outs for assessing developmental toxicity namely morphometrics and cytotoxicity assays, such as ATP assay and Caspase 3/7 antibody staining. b) Fluorescence montage image of the well with XEn/EpiCs; inlet: zoomed in XEn/EpiC with Epi (Hoechst), XEn (Hoechst+ Gata6:mVenus reporter), and PAC (Phalloidin) (Table 03). c) Classification of different phenotypes based on the developmental timeline they represent and the tissues they form and organize. d) Automated image analysis pipeline to derive the morphometrics of XEn/EpiCs, which were used to perform a supervised machine learning algorithm to measure the yield of different phenotypes observed in the images. e) Yield % of the five phenotypes observed upon exposure to non‐teratogenic compounds (legend in [c]). f) Yield% of the five phenotypes observed upon exposure to teratogenic compounds. The asterisks represent the statistically significant change in the yield% of different XEn/EpiC variants. g,h) Automated measurement of the area of XEn/EpiCs upon exposure to non‐teratogenic and teratogenic compounds, respectively; each dot represents one XEn/EpiC. Data are mean ± s.d. obtained from *n* = 3 wells, with each well containing ≈ 165 structures. All statistical hypothesis testing was done using Dunnett's test; ∗ represents *P* ≤ 0.05, ∗∗ represents *P* < 0.01, ∗∗∗ represents *P* < 0.001, and ∗∗∗∗ represents *P* < 0.0001 (one‐way ANOVA with Dunnett's post‐test).

To initiate the formation of XEn/EpiCs within microwell arrays, mouse embryonic stem cells (mES cells) were seeded into 96‐well screening plates containing arrays of thermoformed microwells of 300 µm diameter, at a density of 18–24 cells per microwell. For the first 24 h, the cells were exposed to a chemically defined induction medium to enable the specification of epiblast (Epi) and extraembryonic endoderm (XEn) (**Figure** **1**a). After 24 h, the induction medium was replaced with a basal medium without added serum or signaling molecules. From 48 to 72 h, the structures were exposed to the treatment compounds at three concentrations, with the control condition treated with 0.1% DMSO or water to account for the solvent in all the treatment conditions (Figure 1a). The XEn/EpiCs were fully developed at 120 h (Figure 1b), which is the endpoint of the experiment to conduct different qualitative and quantitative validations.

**Figure 2:**
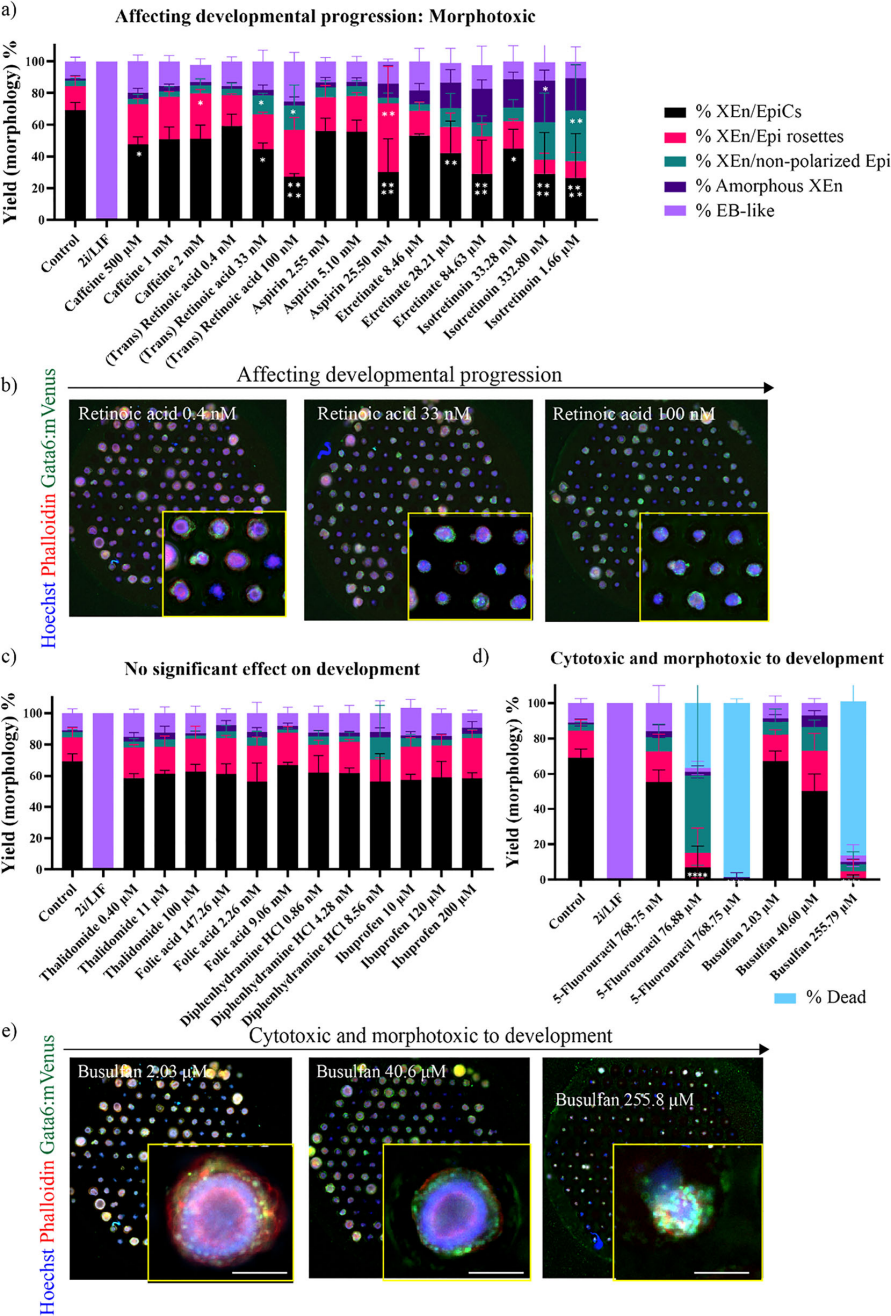
Other notable effects of compounds on the development of XEn/EpiCs: a) Yield% of the five phenotypes observed for compounds affecting developmental progression of XEn/EpiCs. b) Fluorescence montage image of the well with structures obtained after retinoic acid treatment at 0.4 nm, 33 nm, and 100 nm. (Inset) zoomed in wells showing the morphologies of structures observed in each treatment condition. c) Yield % of the five phenotypes observed for compounds causing no significant effect on the yield of XEn/EpiCs formation. d) Yield% of the five phenotypes observed for compounds showing a very toxic effect. e) Fluorescence montage image of the well with structures obtained after Busulfan treatment at 2.03 µm, 40.6 µm, and 255.8 µm. The images were brightened for viewing purposes only. The raw images were used for automated analysis. Data are mean ± s.d. obtained from *n* = 3 wells, with each well containing ≈ 165 structures. Scale bar: 100 µm; All statistical hypothesis testing was done using Dunnett's test; ∗ represents *P* ≤ 0.05, ∗∗ represents *P* < 0.01, ∗∗∗ represents *P* < 0.001, and ∗∗∗∗ represents *P* < 0.0001 (one‐way ANOVA with Dunnett's post‐test).

In previous work, we developed an automated image analysis pipeline using CellProfiler software to identify the structures observed based on morphometrics, such as the area, shape, and intensity profiles; and performed supervised machine learning using CellProfiler Analyst to classify the diverse morphologies observed in our stem cell‐based embryo models and to quantify key morphometric parameters.^[^ ^14b^ ^]^ This system enabled us to systematically evaluate a library of teratogens and categorize their effects into four major groups: i) compounds causing a dose‐dependent (gradient) effect (Figure 1e,f), ii) compounds that disrupt developmental progression through morphological changes (morphotoxic) (**Figure** **2**a,b), iii) compounds causing no significant effect on XEn/EpiCs development (Figure 2c), and iv) compounds inducing high cytotoxicity and morphotoxicity (Figure 2d,e). The formation efficiency (yield) for each type of morphology was scored and compared between conditions (treatments and controls) to identify potential effects on successive morphogenetic key events during development. A multi‐parametric phase‐diagram analysis performed on the yield of XEn/EpiCs versus the area of XEn/EpiCs due to compound exposure is shown in Figure S1, Supporting Information. The different compounds grouped for their respective effects are plotted in Figures S2 and S3, Supporting Information.

Some of the compounds of non‐teratogenic origin (such as ascorbic acid, penicillin, indapamide, and thiamine) showed a slight but significant reduction in the efficiency of XEn/EpiCs formation at the highest dose (Figure 1e); while, some compounds of known teratogenic origin (such as valproic acid, dexamethasone, bosentan, and carbamazepine) showed a slight decrease in the ratio of XEn/EpiCs to other morphologies (Figure 1f), together indicating a gradient (dose‐dependent) effect. The significant change (indicated by the asterisks in the figures) denotes a statistically significant alteration in the yields specifically of the XEn/EpiC morphology class.

For example, the yield% of XEn/EpiCs exposed to penicillin G at 63 µm (low), 1 mm (medium), and 2 mm (high) were 54% (*p* ≤ 0.05), 52% (*p* < 0.01), and 49% (*p* < 0.0001), respectively, in comparison to 72% in control (Figure 1d). Meanwhile, the ratios of XEn/non‐polarized Epi structures increased from 4% at the low and medium doses to 10% at the high dose (Figure 1e). A similar trend was observed for a high dose treatment of Thiamine (Figure 1e). Along the same lines, the reduction in the yield of XEn/EpiCs due to ascorbic acid was accompanied by a reduction in the yield of XEn/Epi rosettes from 18% in the medium dose to 12% at high dose. This was balanced by an increase in the yield of XEn/non‐polarized Epi from 2% and 5% at low and medium doses, respectively, to 18% at the high dose (Figure 1e). It has been shown from several other studies that a series of well‐controlled events in the epiblast, namely the vectoral fluid transport, cytoplasmic vesicle release into the intercellular space, integrin‐mediated adhesion, and water influx into the epiblast center, facilitated lumen expansion.^[^ ^18^ ^]^ The observations of an increased ratio of XEn/non‐polarized Epi structures in our study could potentially indicate the effect of compounds on epiblast polarization and lumenogenesis.

Similarly, the %XEn/EpiCs treated with dexamethasone at concentrations 10 nm, 1 µm, and 100 µm significantly reduced by 60%, 58% (*p* ≤ 0.05), and 55% (*p* < 0.01), respectively (Figure 1e). The high dose of dexamethasone also showed an increase in the ratio of amorphous XEn with 6% compared to 2% at the lower dose, and EB‐like structures to 18% compared to 14% at the lower dose (Figure 1f). This trend was also observed in the treatment with bosentan and carbamazepine, both of which have been shown to induce a dose‐dependent teratogenicity on human in vitro micropatterned pluripotent stem cells by Xing, J., et al., 2017.^[^ ^10b^ ^]^ The other compounds in the library that showed milder effects are shown in Figure S4, Supporting Information.

Further quantification of the overall area of XEn/EpiCs exposed to different treatment conditions showed no significant change in the size (Figure 1g,h) with an exception for treatment with 4 µm valproic acid, which showed a significant reduction in the size with 5000 µm^2^ (*p* < 0.0001) in comparison to 7000 µm^2^ in control (Figure 1h).

Overall, four out of ten compounds from the non‐teratogenic class, namely ascorbic acid, penicillin G, indapamide, and thiamine, showed a dose‐dependent effect at higher doses, without a significant change in the size of XEn/EpiCs. The remaining compounds from the non‐teratogenic class displayed a more pronounced effect, which is discussed in the next section. This shows that compounds at commonly exposed dosages, in line with previous findings from standardized toxicological assays, do not appear to affect cell viability (as seen from the size of XEn/EpiCs) or morphogenesis, but only mildly affect the efficiency of embryonic structures to progress in development. Together, these results show a novel perspective to the known findings for some compounds.

### 2.3 Other Notable Effects of Compounds on the Development of XEn/EpiCs

In the same study, commonly used compounds, such as all‐trans retinoic acid (a metabolic derivative from vitamin A), caffeine, and aspirin, more severely impacted XEn/EpiCs formation when exposed from 48 to 72 h of culture. With increasing dosage, these compounds showed increased morphotoxicity (morphological effect affecting developmental progression), evident from the higher yield of less developed XEn/EpiC morphologies. For instance, treatment with caffeine, retinoic acid, and aspirin led to higher yield of XEn/Epi rosettes; retinoic acid, etretinate, and isotretinoin led to a higher yield of XEn/non‐polarized Epi structures; retinoic acid and aspirin led to a higher yield of EB‐like structures; and etretinate, and isotretinoin led to a higher yield of amorphous XEn structures (Figure 2a,b). Exposure to retinoic acid at 0.4 nm (low), 33 nm (medium), and 100 nm (high) dosages, showed a steep reducing trend in the yield of XEn/EpiCs (Figure 2b) with 58%, 43% (*p* ≤ 0.05), and 28% (*p* < 0.0001), respectively, compared to 72% in control (Figure 2a). Meanwhile, the yield of XEn/non‐polarized Epi significantly increased, with 14% in the medium and high doses compared to 4% in the control (Figure 2a,b). Similarly, the exposure to caffeine showed a reduction in the yield of XEn/EpiCs between the three doses from low to high dose with 48%, 50%, and 52% (*p* ≤ 0.05), respectively, compared to 72% in the control (Figure 2a). Synthetic retinoid derivatives, such as etretinate and isotretinoin, also showed a dose‐dependent effect on the formation of XEn/EpiCs, significantly reducing the yield and displaying a higher ratio of earlier XEn/EpiC stages. Especially, 332.8 µm isotretinoin led to a higher ratio of XEn/non‐polarized Epi and Amorphous XEn phenotypes. This could be a result of a failed or delayed Epi polarization, XEn epithelialization, and PAC formation (Figure 2a).

Compounds, such as thalidomide, folic acid, diphenhydramine, and ibuprofen, showed a similar yield of XEn/EpiCs as the control with no significant effect on the morphology (Figure 2c). For instance, the treatment with folic acid at 147.26 µm (low), 2.26 mm (medium), and 9.06 mm (high) dosages showed a XEn/EpiC yield of 60%, 58%, and 68%, respectively in comparison to 72% in the control (Figure 2c). However, the known teratogenic compound, thalidomide, did not cause a significant reduction in the yield of XEn/EpiCs; instead, a slight but not‐significant increase was noticed at the highest dose of 100 µm (62.63%) in comparison to 0.4 µm (58.64%) (Figure 2c). This observation should be attributed to the known mechanism of action of thalidomide on a later stage of embryogenesis; while, the XEn/EpiCs represent a peri‐implantation stage. Further, this observation should be confirmed using a higher dose of the compound to identify the lowest dose at which it affects XEn/EpiC development. Taking these observations together, we observed that these compounds had no detrimental effect on XEn/EpiCs development for the concentrations tested here.

The compounds 5‐fluorouracil and busulfan, which are chemotherapeutic drugs, showed higher developmental toxicity with increasing doses (Figure 2d). Treatment with 5‐fluorouracil at 768.75 nm (low), 76.875 nm (medium), and 768.75 µm (high) dosages showed a pronounced reduction in the yield of XEn/EpiCs with 55%, 20%, and 0%, respectively (Figure 2d). The ratio of XEn/non‐polarized Epi structures was higher when exposed to 76.875 nm (medium) dose with 32% compared to 4% in the control. It was also observed that the medium and high doses of 5‐fluorouracil induced cytotoxicity in the culture with 36.6% and 98.58% cell death, which was visible by a total reduction in the sizes and disintegration of XEn/EpiC structures (Figure 2d). In a similar case, the fluorescence montage images of structures upon busulfan treatment showed 87.4% cell death at the highest dose and a developmental halt with 12.63% of the structures forming earlier XEn/EpiC variants, affecting polarization and expansion of XEn and Epi (Figure 2e). This could be explained by the mechanism of action of these compounds from in vivo and in vitro studies, whereby they have been shown to induce cell cycle arrest and apoptosis.^[^ ^19^ ^]^ This observation shows that compounds such as 5‐fluorouracil and busulfan are both cytotoxic as well as morphotoxic at high doses (Figure 2d).

Overall, we observed that compounds, such as retinoic acid, etretinate, isotretinoin, and aspirin, affected the developmental progression of XEn/EpiCs at higher doses, resulting in higher yield of XEn/Epi rosettes or XEn/non‐polarized Epi. Moreover, treatment with caffeine affected the developmental progression of XEn/EpiCs at all doses. Thus, these compounds exerted a morphotoxic effect on the XEn/EpiCs development without affecting its size. In addition, we also found that some chemotherapeutic or apoptosis‐inducing drugs, such as busulfan and 5‐fluorouracil, caused a developmental halt at the high doses.

This approach highlights the nuanced mechanisms of developmental disruption by certain compounds, emphasizing the importance of morphological assessment in toxicity studies. It is to be noted that these indications also need follow‐up studies to show the relevance of these findings at later stages of development.

### 2.4 Assessing Morphotoxicity Through Image‐Based Qualitative And Quantitative Read‐Outs

**Figure 3:**
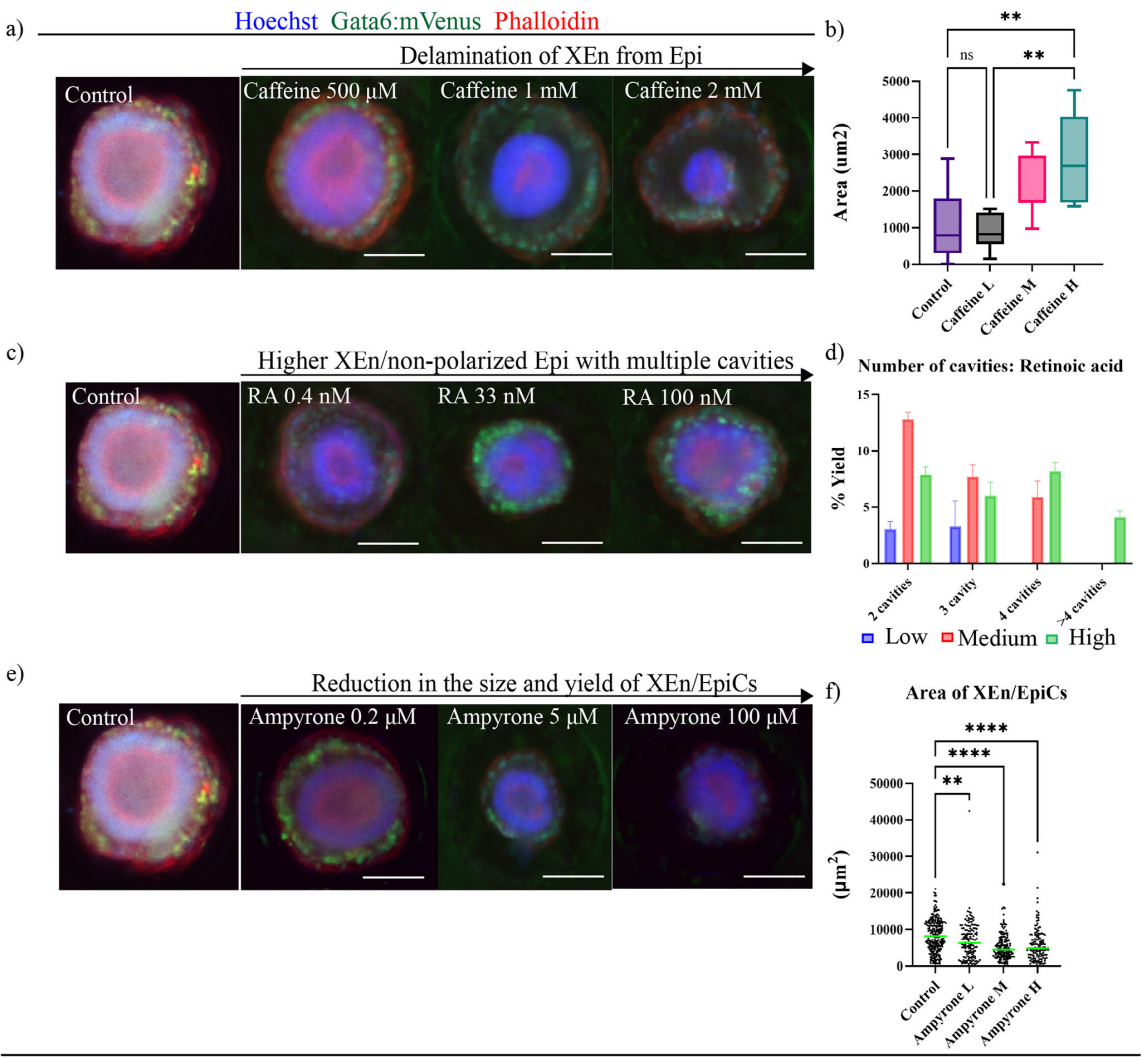
Automated morphometric measurements of the different types of developmental toxicity effect: a) zoomed in fluorescence images of single XEn/EpiCs at increasing doses of caffeine. b) Measurement of the delamination area among the high, medium, and low doses of caffeine by subtracting the area of epiblast from the inner area of XEn. c) Zoomed in fluorescence images of single XEn/EpiCs at increasing doses of retinoic acid. d) Quantification of the number of cavities formed in the XEn/non‐polarized Epi structures with 2, 3, 4, and > 4 cavities per structure. e) Zoomed in fluorescence images of single XEn/EpiCs at increasing doses of ampyrone. f) Automated quantification of the size of XEn/EpiCs in all treatment conditions. Data are mean ± s.d. obtained from *n* = 3 wells, with each well containing ≈ 165 structures. Scale bar: 100 µm. All statistical hypothesis testing was done using Dunnett's test; ∗ represents *P* ≤ 0.05, ∗∗ represents *P* < 0.01, ∗∗∗ represents *P* < 0.001, and ∗∗∗∗ represents *P* < 0.0001 (one‐way ANOVA with Dunnett's post‐test).

Assessment of developmental toxicity through morphological features can elucidate the mechanisms of compounds that do not broadly disrupt development but target specific morphogenetic events. To observe if the compounds in our library caused a morphotoxic effect, we used the automated yield measurements from CellProfiler Analyst (CPA) that were generated earlier to identify the ratio of the different phenotypes. The supervised machine learning classifier provided information on the rates of developmental progression based on the morphological milestones along the embryonic developmental continuum. The morphotoxic effect was then identified from the effect of compounds that showed a higher proportion of earlier stages of XEn/EpiCs or displayed a variation in the area measurements. We found that compounds, such as caffeine, busulfan, dexamethasone, and valproic acid, led to an increase in the delamination of the XEn layer from the epiblast, displaying a more detached structure compared to the more compact XEn/EpiCs in control. The exposure to caffeine at the medium and high doses, namely, 1 mm and 2 mm, led to an increased occurrence of delamination in the XEn/EpiCs, with the 2 mm dose causing the most significant morphological effect (**Figure** **3**a). Quantification of the delamination area, that is, the space between XEn and Epi, using ImageJ to manually measure and subtract the Epi area from the inner XEn area, showed a significantly higher increase with 2600 µm^2^ at 2mm (high) dose compared to 800 µm^2^ at 500 µm (low) of caffeine (Figure 3b).

To characterize the delaminated structures in an automated set‐up, a CPA pipeline was created to perform supervised machine learning for delaminated vs. non‐delaminated structures. The resulting algorithm scored all the treatment conditions and measured the yield of delaminated structures per treatment condition. It was observed that there was a significantly higher ratio of delaminated structures upon exposure to caffeine and busulfan, with 38% and 50% respectively, compared to 16% in the control (Figure S5, Supporting Information). While the treatment with valproic acid and dexamethasone also showed an increased yield with 24% with the high doses, no statistical significance was observed compared to the control (Figure S5, Supporting Information).

Exposure to retinoic acid (RA) was found to significantly reduce the yield of XEn/EpiCs but increase the yield of XEn/non‐polarized Epi structures (Figure 2a). Upon further investigation of the morphology of the XEn/non‐polarized Epi structures after RA exposure, XEn epithelialization appeared to occur normally; however, the non‐polarized Epi had formed multiple, dispersed cavities when exposed to medium and high doses of RA (Figure 3c). This suggests that Epi polarization and PAC formation were affected, possibly due to the morphotoxic effect of RA. Quantification of the number of cavities in the structures from three doses showed that at the lowest dose (0.4 nm), 45% of the structures formed single cavities and 12% formed no cavities, corresponding to the XEn/EpiCs and XEn/non‐polarized Epi structures (Figure S6, Supporting Information). In addition, 3% of structures formed two or three cavities each. A majority (45%) of structures exposed to medium doses (33 nm) of RA resulted in XEn/non‐polarized Epi structures with no cavity (Figure S6, Supporting Information). However, interestingly, the medium dose of RA also caused a high number of structures with two, three, and four cavities, with 13% (≈16 structures) with two cavities; while, 8% and 6% of structures developed three and four cavities, respectively (Figure 3d). Similarly, the highest dose of 100 nm RA developed several structures with two, three, four, and five cavities with 7%, 6%, 8%, and 4%, respectively (Figure 3d).

Some compounds, such as ampyrone, did not show a significant effect on the yield of XEn/EpiCs, but further investigation of the morphometric data from CellProfiler showed a significant reduction in the size of XEn/EpiCs (Figure 3e), measured as a reduction in the projected area of XEn/EpiCs from 9000 µm^2^ to 7000 µm^2^, 5000 µm^2^, and 5000 µm^2^ in low, medium, and high doses, respectively (Figure 3f).

Overall, the observation of the different features in the structures showed a potential morphotoxic effect on the formation of XEn/EpiCs with or without necessarily having an immediate cytotoxic effect. These compounds included retinoic acid, caffeine, busulfan, valproic acid, dexamethasone, and ampyrone. Further extensive experimentation in the future should focus on the developmental progression of these XEn/EpiCs for a longer duration to identify the effect of these morphological changes on the later developmental stages. Together, these results emphasize the added value of including morphotoxicity metrics in high‐throughput DART assays complementary to traditional cytotoxicity assays, which would provide a more holistic view of the effects of certain compounds on development.

### 2.5 Teratogen Dose Response Curves to Assess the Range of Developmental Toxicity

**Figure 4:**
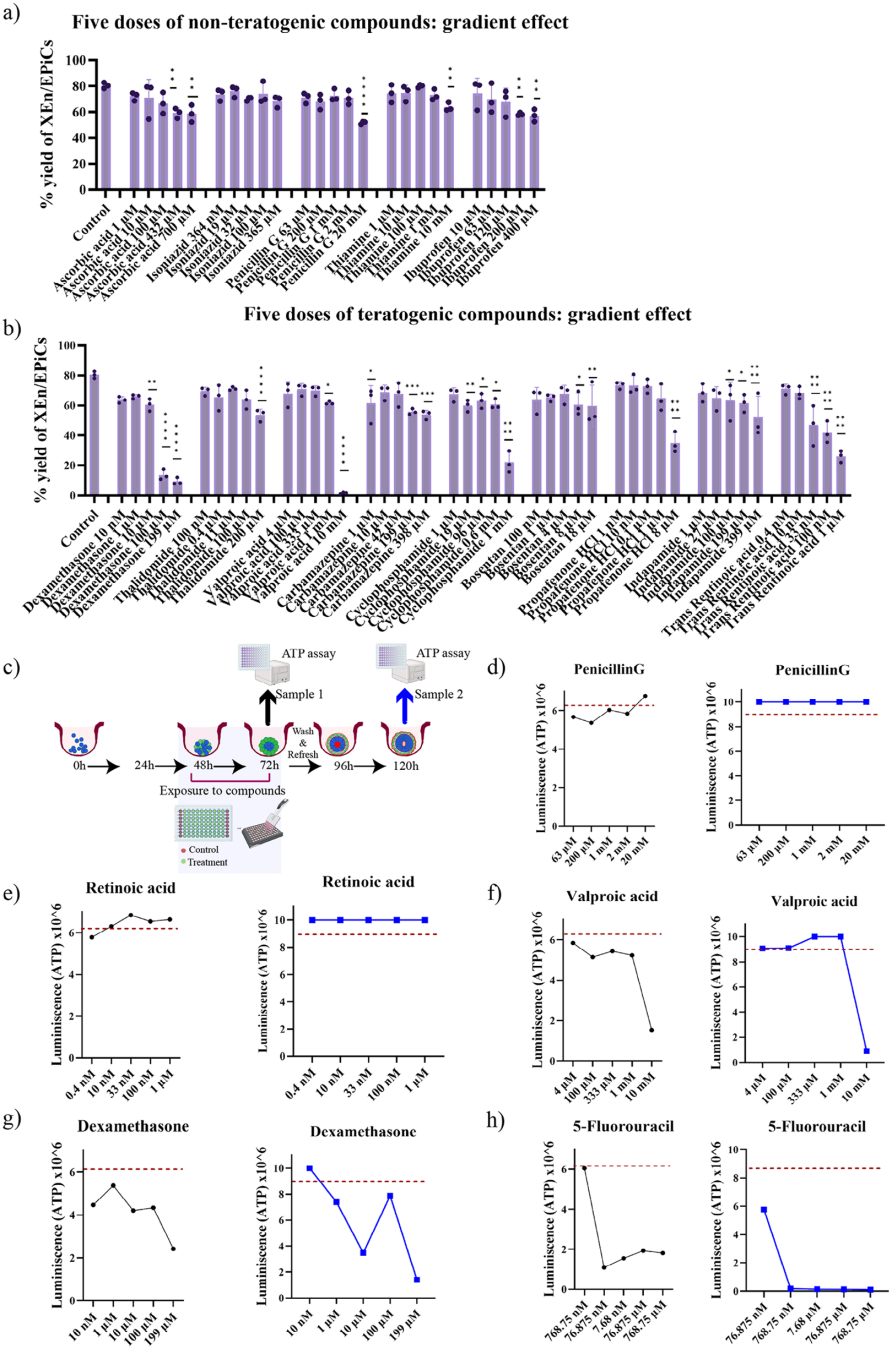
Screening with five concentrations of teratogens to assess the range of cytotoxicity: Yield% of XEn/EpiCs after exposure to a) non‐teratogenic compounds and b) teratogenic compounds from 48 to 72 h. c) Experimental design for CellTiter Glo 3D ATP assay on the treated and control samples at 72 and 120 h of development; ATP assay using CellTiterGlo 3D detecting the levels of ATP released by the living cells when exposed to d) penicillin G, e) retinoic acid, f) valproic acid, g) dexamethasone, and h) 5‐Fluorouracil at five increasing doses. The black line indicates the levels of ATP from sample 1 collected at 72 h; the blue line shows the levels of ATP from sample 2 collected at 120 h, after the removal and washing of compounds; and the red dotted line represents the control ATP levels. Each point represents a pooled sample from three biological replicates (*n* = 3), that is, from three individual wells of a 96‐well plate, containing ≈ 165 structures/well. The suspension containing the structures was transferred to an opaque‐walled 96‐well plate to read the luminescence accurately. An integration time of 0.25–1 s per well was used as a guideline for detection based on the manufacturer's instructions.

From the initial screen with three concentrations, the highest and lowest doses required to elicit an effect on XEn/EpiCs development were identified. The next step was to extend the limit or add intermediate concentrations to create a more comprehensive picture of the morphological effect caused by the compound. For this purpose, compounds that caused a gradient (dose‐dependent) effect, affected developmental progression, and those causing high cytotoxicity were chosen to run a follow‐up screen with five doses. By observing the yield % of different morphologies in each treatment condition using the automated machine learning pipeline, some non‐teratogenic compounds, such as isoniazid, penicillin, and ibuprofen, still showed a gradient effect on the development of XEn/EpiCs, with the highest dose reducing the occurrence but not significantly affecting its formation, displaying ≈ 60% efficiency (**Figure** **4**a).

For instance, treatment with 63 µm, 200 µm, 1 mm, 2 mm, and 20 mm of penicillin G showed a XEn/EpiCs % of 72%, 70%, 72%, 71%, and 62% respectively, showing that the highest dose of the compound significantly affected the development of XEn/EpiCs compared to the control with 79%, but still less drastic (Figure 4a). Similarly, the addition of ibuprofen at high doses of 200 and 400 µm showed a significant reduction in the yield of XEn/EpiCs by 60% in comparison to the control (Figure 4a).

In contrast, treatment with teratogenic compounds, such as dexamethasone, valproic acid, carbamazepine, cyclophosphamide, bosentan, propafenone HCl, indapamide, and trans‐retinoic acid, showed a drastic cytotoxic effect upon exposure at higher doses (Figure 4b). Specifically, the highest chosen dose of the compound affected the formation of XEn/EpiCs significantly. In the case of dexamethasone, at 10 nm, 1 µm, 10 µm, 100 µm, and 200 µm concentrations, the yield of XEn/EpiCs dropped dramatically with 64%, 66%, 60%, 15%, and 10%, respectively (Figure 4b). Treatment with trans‐retinoic acid showed a progressively increasing toxicity with increasing dosage, with the lowest doses, namely 0.4 nm and 10 nm showing 70% and 68% of XEn/EpiCs respectively; while the higher doses, namely 33 nm, 100 nm, and 1 µm dosage showed 42%, 38%, and 36%, respectively of XEn/EpiCs formation (Figure 4b).

Together, from the exposure of different compounds at five concentrations, we observed an interesting trend in the effect on development. These results helped determine the doses at which there is a morphological effect on development and the doses at which there was maximum developmental toxicity.

To quantify the level of cytotoxicity caused by the exposure to toxic compounds and correlate it to the observations of the morphological changes, the levels of ATP in each condition were measured using the CellTiter Glo 3D assay kit (Promega), whereby ATP levels indicated the number of viable cells. The sample collection and measurement were performed at two time points; the first samples were collected 24 h after exposure to compounds (48–72 h), after which the treatment compounds were removed and refreshed with plain medium. The second samples were collected 48 h after the removal of compounds (120 h) (Figure 4c). The two time points (samples 1 and 2) were chosen to identify the immediate effect of the compound exposure to the structures (acute exposure), as well as the ability of the cells to catch up with the developmental timeline upon removal of the compounds (chronic effect), to identify if the toxicity could be reversed. It was observed that treatment with penicillin G caused a decrease in the ATP levels at lower doses of 63 µm, 200 µm, 1 mm, and 2 mm with 5.6, 5.3, 6.0, and 5.8 × 10^6^ U of luminescence, respectively, relative to 6.2 × 10^6^ U in the control (red line) (Figure 4d). This could be due to the progressively reduced yield of XEn/EpiCs with increasing doses. Interestingly, the levels of ATP released at the highest dose of 20 mm increased to 6.7 × 10^6^ U when measured 24 h after treatment (Figure 4d). Meanwhile, the ATP levels remained constant at all doses, relative to the control, when measured at 120 h (sample 2), implying no cytotoxic effect on development upon removal of the compound (Figure 4d). This increase in ATP levels 24 h after exposure to a very high dose of penicillin could be due to a shift in the cellular metabolism toward high ATP release, an effect also seen in antibiotic‐treated bacterial cells.^[^ ^20^ ^]^ A unique observation was the 24 h treatment with retinoic acid (RA), which showed an increase in the ATP levels, with increasing dosage, relative to the control (Figure 4e). Similar to the high dose of penicillin, this could be due to a metabolic shift to oxidative phosphorylation induced by RA, immediately after exposure, at higher doses. Interestingly, the recovery at 120 h (sample 2) displayed uniform ATP levels, resembling the control, indicating the reversal of the effects of RA upon removal of the compound. This shows that despite causing a morphotoxic effect, affecting the developmental progression, as observed in Figure 3c, there was no significant cytotoxic effect upon removal of the compound at the chosen dosages (Figure 4e).

In the case of valproic acid, there was a significant drop in the ATP levels relative to the baseline control as the concentration increased, with the most significant drop of 1.5 × 10^6^ U when exposed to a 10 mm dose (Figure 4f). The measurement of sample 2 showed a complete recovery in the cell number except in the case of 10 mm (highest) dose where the ATP levels seemed to drop significantly to 0.9 × 10^6^ U. Together, this shows the irreversible cytotoxic effect of valproic acid on XEn/EpiC development at 10 mm dose that failed to rescue the effects despite the removal of the compound (Figure 4f). Treatment with dexamethasone displayed reduced levels of ATP, with the highest dose (199 µm) treatment displaying 2.4 × 10^6^ U of ATP relative to the baseline (Figure 4g). The recovery in cell numbers and development in sample 2 at a 10 nm dose showed 9.9 × 10^6^ U of ATP, similar levels to the control. However, the higher doses showed reduced levels of ATP, with the sample 2 at the highest dose displaying 1.4 × 10^6^ U of ATP (Figure 4g). It was interesting to note that dexamethasone at a 100 µm dose, in both sample 1 and sample 2, had a relatively higher level of ATP compared to a 10 µm dose. (Figure 4g). This unique effect is attributed to dexamethasone's non‐monotonic dose‐response relationships when treated on stem cells. For instance, dexamethasone has been widely accepted to enhance the differentiation of mesenchymal stem cells toward osteogenic, chondrogenic, and adipogenic differentiation.^[^ ^21^ ^]^ However, depending on the dose, the differentiation can be steered preferentially toward adipogenic rather than osteogenic differentiation^[^ ^22^ ^]^ at 10^─7^ mol L^─1^; while, at a lower dose of 10^─8^ mol L^─^, dexamethasone could maintain the stemness and proliferation of MSCs.^[^ ^21^ ^]^ In our study, the ATP levels showed a peak at 100 µm dose, possibly indicating a threshold dosage, where dexamethasone could have an inhibitory effect on differentiation, also accounting for the reduced yield of XEn/EpiCs at this dose. The anti‐cancer drug, 5‐fluorouracil, displayed a severe cytotoxicity beyond 76.876 nm, dose with the ATP levels dropping to 1.0 × 10^6^ U, and the recovery of these structures at 120 h in sample 2 also showed very low levels of ATP (Figure 4h). This is consistent with the previously observed yield measurements using automated quantification about the cytotoxic and morphotoxic effect of 5‐fluorouracil (Figure 2d).

These findings suggest that compounds, such as retinoic acid, which did not show a cytotoxic effect at the high doses of exposure at 120 h, showed a morphotoxic effect as shown in Figure 3c. Meanwhile, it was also interesting to find that the morphotoxic doses of dexamethasone and valproic acid, which showed a significant reduction in the yield of XEn/EpiCs, also showed an increased cytotoxic effect. This again emphasizes the significance of morphometric read‐outs complementing cytotoxicity studies to fully understand the compounds’ implications on development.

### 2.6 Characterization of Cell Death With Morphogenetic Read‐Out as a Measure of Developmental Toxicity

**Figure 5:**
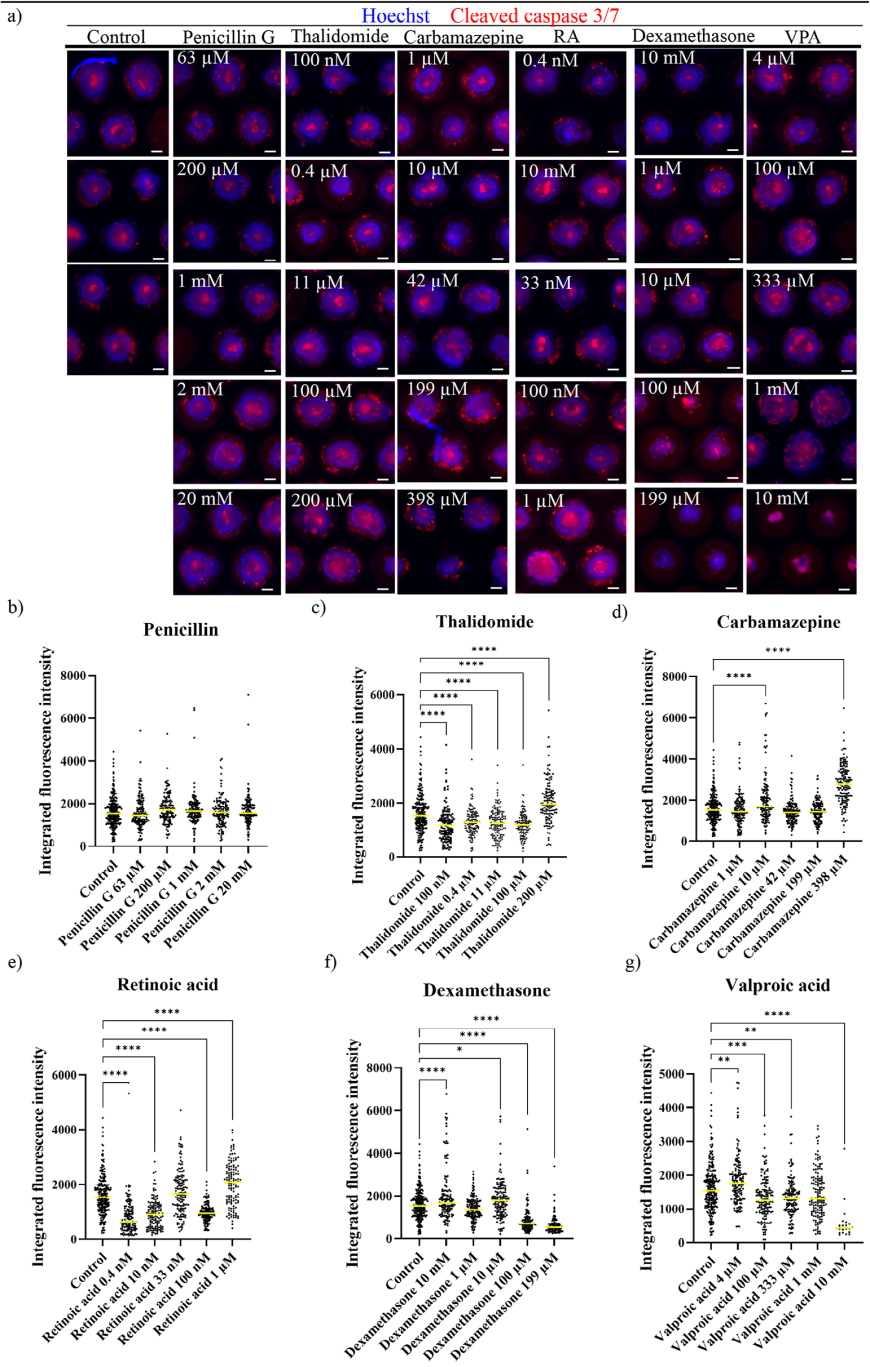
Characterization of cell death with morphogenetic read‐out as a measure of developmental toxicity: a) fluorescence images of 120 h XEn/EpiCs stained for cell death marker cleaved Caspase 3/7 and Hoechst in control and when treated with penicillin G, thalidomide, carbamazepine, retinoic acid, dexamethasone, and valproic acid, at five doses. Integrated intensity measurement of the objects when treated with b) penicillin G, c) thalidomide, d) retinoic acid, e) carbamazepine, f) dexamethasone, and g) valproic acid. Data are mean ± s.d. Scale bar: 100 µm. All statistical hypothesis testing was done using Dunnett's test. ∗ represents *P* ≤ 0.05, ∗∗ represents *P* < 0.01, ∗∗∗ represents *P* < 0.001, and ∗∗∗∗ represents *P* < 0.0001 (one‐way ANOVA with Dunnett's post‐test).

From the treatment with the compound at five concentrations, it was observed that the highest doses caused a very drastic reduction in the yield of formation of XEn/EpiCs. The most common assumption for this effect would be that the compounds cause cell death, thereby resulting in the drop in % XEn/EpiCs. To qualitatively assess the nature of cell death in these structures, the 120 h XEn/EpiCs in each treatment condition, for all five concentrations, and the control were stained with cleaved Caspase 3/7 antibody, a commonly used marker to verify cell death with its increased expression in late apoptotic and dying cells. The cells were also stained for Hoechst (nuclei) (**Figure** **5**a) (Table 03).

It was observed that the XEn/EpiCs formed in the control condition showed a cluster of Caspase 3/7^+^ cells in the center of the structure within the pro‐amniotic cavity. From previous studies, it has been shown that at E4.5, the epiblast receives an integrin‐mediated polarization cue from the basement membrane secreted by the surrounding visceral endoderm, water influx through osmotic gradients, and E‐Cadherin/Podocalyxin‐exclusive apical‐basal polarity, leading up to the expansion of the pro‐amniotic cavity (PAC).^[^ ^18^ ^]^ However, due to the lack of external serum or matrix supplementation in the medium of the in vitro cultured XEn/EpiCs, the lumenogenesis involves increased apoptosis inside the cavity.^[^ ^23^ ^]^ Hence, the XEn/EpiCs in all treatment conditions showed a Caspase 3/7^+^ cluster of cells inside the PAC. However, when compared to the control, the treatment conditions showed a different level of cell death depending on the concentration.

In the case of penicillin G, the ratio of Caspase 3/7^+^ cells in the lumen of XEn/EpiCs increased with increasing dosages and was more pronounced at a 20 mm dose (Figure 5a). The level of Caspase 3/7 expression in each object (per well) was calculated using the automated pipeline, measuring the integrated intensity over space. Automated measurement of the integrated intensity in the structures under the different treatment doses of penicillin showed no significant increase in the Caspase 3/7 expression compared to the control (Figure 5b).

The exposure to thalidomide did not show a drastic reduction in the yield of XEn/EpiCs (Figure 5a). However, at the highest dose of 200 µm, there was an effect on the morphology (Figure 5a) as well as an increased Caspase 3/7 intensity (Figure 5c). Similar to thalidomide treatment, exposure to carbamazepine showed a comparatively higher yield of XEn/EpiCs formation at the lower doses and a slightly reduced yield at the higher doses (Figure 4b). However, immunostaining of structures revealed a higher Caspase 3/7 expression at the highest dose of 398 µm (Figure 5d) with a Caspase 3/7+ population in both PAC and XEn compartments, validated by the increased intensity compared to the control (Figure 5d).

Treatment with retinoic acid showed interesting effects on the XEn/EpiCs development. The intensity of Caspase 3/7 seemed minimal at 0.4 nm and 10 mm concentration, proportional to the previous observations (Figure 5a). However, the effects were more pronounced from 10 nm concentration, significantly causing morphotoxicity as well as cytotoxicity with the treatment with the highest dose of 1 µm retinoic acid forming XEn/non‐polarized Epi structures (Figure 5a), displaying an increased Caspase 3/7 intensity (Figure 5e). Exposure to dexamethasone showed a morphological reduction when exposed to a 100 µm dose and beyond (Figure 5a), forming more EB‐like or amorphous XEn structures instead of XEn/EpiCs. This was also seen from the reduced integrated intensity at 100 and 199 µm of dexamethasone (Figure 5f). This shows that treatment with dexamethasone affected XEn epithelialization and Epi polarization (Figure 4a). Similar to dexamethasone, the treatment with valproic acid increased the ratio of cell death in the PAC with increasing dosages (Figure 5a). However, at the highest dose of 10 mm valproic acid, the drastic cell death was accompanied by a stunted morphology with no formation of XEn/EpiCs in this condition, forming only EB‐like structures (Figure 4a). This was also corroborated with the measurement of integrated intensity of Caspase 3/7 expression, which showed a significant increase with increasing doses (Figure 5g).

Measurement of the radial intensity distribution of Caspase 3/7 fluorescence allowed the visualization of the area of cell death in different radial bins within XEn/EpiCs (Figure S7, Supporting Information), where each bin corresponds to XEn, Epi, or PAC. This way, the cell death, represented by Caspase 3/7 positive cells, could be delineated per compartment through the quantification of the coefficient of variation (radialCV) of intensity between the different bins (Figure S8, Supporting Information). The observed results for the effect of valproic acid correlated with the previous findings and showed that the radialCV of bins 1, 2, and 3 were significantly increased in the 333 µm and 1 mm doses, indicating high cell death in the PAC and XEn compartment (Figure S8, Supporting Information). Moreover, the 10 mm (highest) dose, which seemed to have caused a high cytotoxic and morphotoxic effect, also showed a highly affected morphology on the schematic of radial intensity distribution (Figure S7, Supporting Information).

Putting all the findings together, we show that the measurement of cytotoxicity can be performed in parallel to morphological assessment within the microwells using an automated image analysis pipeline, directly correlating the morphology to the developmental process that has been affected. We use the pipeline to further quantify the spatial intensity distribution of cell death using a caspase 3/7 staining, providing clues on the effect of valproic acid on the different tissue compartments.

## 3 Discussion

This pilot study demonstrates that XEn/EpiCs cultivated in microwell arrays present a versatile model, enabling robust formation and high‐throughput imaging of embryo‐like structures and facilitating the extraction of extensive morphological and functional data. A direct or indirect exposure to exogenous agents can disrupt mammalian fetal development, making it a high priority to evaluate and validate new and existing drug candidates for developmental toxicity and teratogenicity. A key advantage of XEn/EpiCs, in comparison to other 3D embryo models, is their controlled formation of both epiblast and extraembryonic endodermal tissues, which co‐develop in response to chemical induction through the peri‐implantation stage. Previously, we had shown the robust formation of XEn/EpiCs in a thermoformed microwell platform, enabling the extraction of morphological information from the structures for modulating signaling pathways.^[^ ^14b^ ^]^ Using this system, we assessed a compound library and observed dose‐dependent toxicity effects on XEn/EpiCs, influencing various morphogenetic processes and tissue types.

Traditional toxicity assessments rely on cytotoxicity measurements through microtiter assays and end‐point analyses that evaluate disrupted proliferation, differentiation, and migration. However, developmental toxicity often manifests as morphological changes during early embryogenesis; and thus, relying solely on cytotoxicity or differentiation markers provides an incomplete understanding of a compound's teratogenic potential. A more comprehensive approach is required to evaluate the impact of teratogens on embryonic development fully. Our model's unique feature lies in its ability to automatically detect, extract, and quantify parameters such as area, shape, size, texture, and intensity from each XEn/EpiC, facilitating the classification of observed morphologies and their association with affected developmental events.^[^ ^14b^ ^]^

Teratogens, compounds that cross the maternal barrier during pregnancy, can disrupt the embryonic morphogenetic program with varying degrees of severity. These compounds are traditionally categorized based on their effect, severity, dosage, and validation in animal models. While a more extensive exploration of XEn/EpiC development at later stages, using a broader dose range and treatment window, is required to fully validate XEn/EpiCs as an in vitro model for teratogenicity assessment, this pilot study provides important initial insights.

Our results, as summarized in **Table** **2**, identified minimal to no effect on XEn/EpiC formation following exposure to commonly prescribed drugs such as ibuprofen and folic acid. This follows the known and well‐studied effects of oral folic acid supplementation, which is widely recommended for women before conception and during the first trimester of pregnancy due to its critical role in supporting neural tube development.^[^ ^24^ ^]^ Exposure to compounds that have not shown a significant teratogenic effect in rodent models, such as thalidomide^[^ ^25^ ^]^ and diphenhydramine HCl (FDA category B),^[^ ^26^ ^]^ also did not affect XEn/EpiCs development in the concentrations tested in this study. These results suggest XEn/EpiCs as a relevant and alternative mouse embryo model that can recapitulate the findings of known compounds and drugs from animal studies.

**Table 2:** Outline of the effects of all compounds: N/A means these compounds were not explored beyond the three‐dose screen in this pilot study.

Effects based three‐dose screen	Toxicity category	Compounds (FDA category)	Cytotoxic dose	Other novel observations
Gradient (dose‐dependent) effect	Non‐teratogen	Ascorbic acid (A)	N/A	N/A
		Penicillin (B)	20 mm	Penicillin caused a spike in the ATP levels at 20 mm dose 24 h after exposure, but the levels were recovered at 120 h
		Thiamine (A)	N/A	N/A
		Indapamide (B)	N/A	N/A
	Teratogen	Valproic acid (D)	10 mm	4 µm VPA led to reduction in size of XEn/EpiCs
		Dexamethasone (C)	199 µm	Dex exposure caused a nonmonotonic spike in the ATP levels at 100 pm
		Bosentan (X)	N/A	N/A
		Carbamazepine (D)	N/A	N/A
		Propafenone HCl (C)	8 µm	N/A
		Ampyrone (N/A)	N/A	Above 5 µm dose, ampyrone led to a reduction in the size of XEn/EpiCs
		Cytosine Arabinoside (C/D)	N/A	N/A
No significant effect on development	Non‐teratogen	Folic acid (A)		N/A
		Ibuprofen (B)	200 µm	N/A
		Diphenhydramine HCl (B)	N/A	N/A
	Teratogen	Thalidomide (X)	100 µm	100 µm Thalidomide affected XEn/EpiCs yield
		Cyclophosphamide (D)	1 mm	N/A
		Isoniazid (C)	N/A	N/A
		Hydroxyurea (D)	N/A	N/A
		Phenytoin (D)	N/A	N/A
Affecting developmental progression (morphotoxic)	Non‐teratogen	Caffeine (N/A)	N/A	2 mm caffeine led to the delamination of XEn from Epi
		Aspirin (C)	N/A	25. 5 mm aspirin led to an increased % of XEn/Epi rosettes
	Teratogen	Retinoic acid (C)	1 pm	100 nm RA developed > two cavities in 28. 3% of structures
		Etretinate (X)	N/A	28. 21 µm etretinate led to increased % of earlier XEn/EpiC variants
		Isotretinoin (X)	N/A	332. 8 µm isotretinoin led to a higher ratio of XEn/non‐polarized Epi and Amorphous XEn structures
Cytotoxic and morphotoxic to development	Teratogen	5‐Fluorouracil (D)	76. 875 µm	The development of XEn/EpiCs seemed to be halted at 48 h, which is the time of addition of the drug
		Busulfan (D)	255. 79 µm	The development of XEn/EpiCs seemed to be halted at 48 h, which is the time of addition of the drug
		Trichlorfon (N/A)	19. 422 µm	N/A

Interestingly, exposure to other non‐teratogenic compounds, namely, ascorbic acid, penicillin, indapamide, and thiamine, demonstrated a dose‐dependent effect, with the highest doses significantly reducing XEn/EpiCs formation (Figure 1e). This aligns with the previously demonstrated dose‐dependent embryotoxicity studies with these compounds, which induced toxicity on rodent embryos at high doses.^[^ ^27^ ^]^ Meanwhile, compounds with well‐documented teratogenic effects, such as valproic acid (VPA),^[^ ^28^ ^]^ dexamethasone,^[^ ^29^ ^]^ bosentan,^[^ ^30^ ^]^ carbamazepine,^[^ ^31^ ^]^ and propafenone HCl^[^ ^32^ ^]^ led to a significant reduction in the yield of XEn/EpiCs at the highest doses. Epi/XEn clusters between 72 and 120 h underwent XEn epithelialization, apical‐basal polarization of the Epi, and lumen formation. By exposing the compounds from 48 to 72 h during XEn/EpiCs development, which mimicked the pre‐implantation window of embryonic development, we could observe the compounds’ effects to disrupt the morphology of the structures, forming earlier‐stage XEn/EpiC variants. For example, VPA exposure from 48 to 72 h could have affected the Epi polarization and lumen formation, thereby forming a higher ratio of XEn/Epi rosettes and XEn/non‐polarized Epi at medium doses and causing extreme cytotoxicity at the highest doses. In the future, combinatorial treatment with folic acid following VPA treatment could potentially rescue the effects of VPA, as seen in human pluripotent stem cell cultures.^[^ ^33^ ^]^

Valproic acid (VPA), a histone deacetylase inhibitor, affects stem cell differentiation dose‐dependently. For instance, it is known to promote stemness as well as differentiation by activating developmental pathways including BMP^[^ ^34^ ^]^ and TGFbeta, depending on the cell type and concentration used.^[^ ^35^ ^]^ We reason that the low doses selectively alter gene expression, possibly reducing pro‐amniotic cavity size by affecting proliferation, apoptosis, or extracellular matrix composition. A recent study, by Zhang et al., 2022 on human pluripotent stem cell‐differentiated neural tube‐like structures, observed a toxic effect due to VPA exposure by inhibiting the lumen formation.^[^ ^33^ ^]^ In our study, we observed that the treatment with 4 µm VPA caused a reduction in the overall size of XEn/EpiCs (Figure 1h), possibly due to VPA altering cytoskeletal function and cell polarization. However, the medium and high doses of VPA did not show this phenotype, albeit causing a reduced yield. We speculate that higher doses cause broader epigenetic changes, potentially activating compensatory mechanisms that offset these effects.

Particularly noteworthy were the compounds that produced significant morphological changes, which we classified as morphotoxic to development (Figures 2 and Figure 3). For instance, caffeine exposure caused delamination of the XEn layer from the Epi, particularly at the highest dose of 2 mm, with a larger delamination area compared to controls (Figure 3a,b). This phenotype mirrors the morphology seen in Nodal knock‐out^[^ ^15^ ^]^ or Nodal inhibited XEn/EpiCs,^[^ ^14b^ ^]^ as well as in conditions with increased Wnt and BMP pathway activation^[^ ^14b^ ^]^ (Figure S9, Supporting Information), suggesting a similar mechanism of action for caffeine. Previous studies have also shown that high doses of caffeine induce severe anomalies in normal mouse embryo development.^[^ ^36^ ^]^ These findings highlight the potential need to reconsider the safety thresholds for caffeine exposure as lower doses may exert subtle yet significant morphological effects on development.

Retinoic acid exposure also yielded intriguing results. At medium and high doses, XEn/EpiCs yield significantly decreased, and a higher percentage of structures remained in an earlier developmental stage, characterized by non‐polarized Epi. Further investigation revealed that these structures frequently contained multiple smaller, disintegrated cavities rather than a single cavity, with the number of disintegrated cavities increasing with higher doses of retinoic acid (Figure 3c,d). These results suggest that high doses of retinoic acid disrupt epiblast polarization and lumenogenesis. This finding aligns with research by Collins and Mao (1999) on the developmental effects of RA,^[^ ^37^ ^]^ echoing observations that fetal RA exposure can lead to severe functional and behavioral abnormalities. The ATP assay after RA exposure showed an increased ATP production when exposed to higher doses of RA, relative to the control, when measured 24 h after compound exposure (Figure 4e). This could be a result of a shift in the metabolic profiles during differentiation in these structures. A relatively recent study on RA‐induced cardiomyocyte differentiation in human embryonic stem cells (hESCs) identified a metabolic maturation characterized by a metabolic shift from glycolysis to oxidative phosphorylation. This led to enhanced ATP production in the cells.^[^ ^38^ ^]^ Although similar studies are lacking in mouse ESCs, it is plausible that RA may influence a similar metabolic activity when exposed at a specific stage and duration during differentiation. Another study identified the effect of passage number on the metabolic profile of extraembryonic endoderm (XEn) cell differentiation, where late passage cells utilized oxidative phosphorylation to derive energy.^[^ ^39^ ^]^ As direct evidence of the metabolic activity in response to RA in mESCs is currently lacking, further research is needed to confirm this effect. A similar effect was also observed when exposed to the highest dose of penicillin at 20 mm.

Expectedly, compounds typically prescribed for cancer treatment, such as busulfan, 5‐fluorouracil, etretinate, and isotretinoin, induced a developmental arrest at 48 h, coinciding with the timing of treatment. No recovery in development was observed following the removal of these compounds (Figure 2). Morphologically, these structures resembled embryoid bodies with Gata6 (mVenus+) cells scattered randomly among Epi cells. This finding suggests that chemotherapeutic agents induce both morphotoxic and cytotoxic effects, emphasizing the importance of using complementary approaches to predict developmental toxicity.

Extending the concentration range of certain compounds to five doses enabled us to identify the dose at which the most severe developmental effects occurred. For valproic acid, dexamethasone, and cyclophosphamide, the yield of XEn/EpiCs dropped to less than 20% at the highest dose (Figure 4). Cytotoxicity was assessed using an ATP‐based microtiter assay at 72 h, shortly after exposure, and at 120 h, following compound removal. Notably, valproic acid, dexamethasone, and 5‐fluorouracil caused irreversible cytotoxicity at the highest doses, with no developmental rescue at 120 h. Interestingly, exposure to a high dose of retinoic acid caused a cytotoxic effect at 24 h, but by 120 h, the structures showed increased ATP release, suggesting enhanced cell proliferation and a recovery in development after compound removal (Figure 4). This finding is consistent with the increased formation of XEn/non‐polarized Epi structures after retinoic acid treatment (Figure 2a).

Future experimentation with a combinatorial exposure of the compounds at different time points and larger concentration ranges could yield a comprehensive dataset to predict the in vivo teratogenicity of the said compounds. An extension of this study would require benchmarking the morphological observations with the other in vitro models and in vivo studies at the different doses, which are currently beyond the scope of a pilot study of this size.

Overall, we observed that compounds with different properties and mechanisms of action had varying impacts on XEn/EpiC development and morphogenesis. Through this screening, we identified several interesting morphotoxic effects of compounds, which were previously only studied in animal models. This approach expands our ability to study developmental toxicity in a more accessible system. We propose that complementing traditional cytotoxicity assays with morphotoxicity assessments offers a more accurate prediction of developmental and reproductive toxicity, potentially achieving outcomes comparable to in vivo experiments and reducing the reliance on animal testing. This method for developmental toxicity assessment can be used as a stand‐alone initial step to short‐list the compounds or used in combination with existing assays to finally minimize the use of animals in testing. Our new platform has the potential to become a powerful tool to perform automated large‐scale toxicological screens and further elucidate the mechanisms affecting development.

### 3.1 Limitations of This System

This pilot study was only tested on a single mouse embryonic stem cell line, of the strain C57BL/6. In the future, this system should be tested on other mouse stem cell lines from the same strain. Further, assessing variability across lines from different strains (e.g., AB2.2, R1/E, CD‐1) would provide deeper insights into its robustness. In addition, extending this system to testing human ESC or iPSC‐derived embryo models using the same library and read‐outs would provide a good comparison of the two species and enable the opportunity to validate the effects across systems. The pipeline for morphometric feature extraction currently recognizes the five primary XEn/EpiC variants. Some of the compounds that induced a pronounced effect should be followed up with a dose‐response observation over time, tracking cell proliferation, and real‐time morphologies, to accurately conclude their toxic effects. Expanding the machine‐learning algorithm to classify unexpected phenotypes, such as by incorporating deep‐learning‐based analysis, would enhance the system's flexibility in detecting novel features, including an excess number of cavities; while, minimizing supervision and error. Performing the screening with a larger concentration range and mapping the morphometric and viability read‐outs systematically to identify the IC50 values of morpho‐ and cytotoxicity is of future interest.

## 4 Experimental Section

### Mouse ES Cell Line

In this study, mES:: Gata6‐h2b‐Venus reporter cell line (mESCs comprising an H2B‐Venus reporter under the regulatory elements of Gata6), which was a kind gift from C. Schröters’ laboratory (Max Planck Institute of Molecular Physiology, Dortmund, Germany), was used.^[^ ^40^ ^]^ The experiments were carried out using cells within passages 10–18. The ES cell line had been tested for mycoplasma. For all the experiments in this article, mES:: Gata6: H2B: Venus reporter line was used.

### Thermoformed Microwells

For the 3D culture of mESCs, thermoformed microwells, STATARRAYs (polystyrene microwell 96‐well plate from 300MICRONS GmbH), were used^[^ ^16^, ^20^ ^]^ Prior to usage, the wells were washed once with 70% ethanol and twice with water. The wells were then treated with anti‐adherence solution (StemCell Technologies) and incubated for ≈ 20 min at room temperature inside the laminar flow hood; and then, washed thrice with PBS. Fresh PBS was added to the wells and stored until further use.

### ES Cell Culture and Reagents

Mouse embryonic stem cells (mESCs) were regularly expanded on a feeder layer of irradiated wildtype (B16/C57) mouse embryonic fibroblasts (mEFs), (T50)—obtained from Erasmus UMC+ iPS core facility—on a 0.15% gelatin‐coated TC plates. For the differentiation to XEn/EpiCs, the mEFs were depleted before seeding into microwells.

The cells were cultured using ES medium composed of: Dulbecco's Modified Eagle's Medium High Glucose (Life Technologies) supplemented with 10% fetal bovine serum (FBS, Greiner), 4 mm Glutamax (Life Technologies), 100 U mL^−1^ penicillin (Life Technologies), 100 mg mL^−1^ streptomycin (Life Technologies), and 10 mm non‐essential amino acids (Life Technologies), and freshly supplemented with 0.1 mm 2‐mercaptoethanol (Life Technologies), 1000 U mL^−1^ leukemia inhibitory factor (LIF, Life Technologies), 3 µm CHIR99021 (GSK3β inhibitor, Axon Medchem), and 1 µm PD0325901 (MEK/ERK inhibitor, Sigma–Aldrich). They were refreshed every alternate day and passaged on the third day. The cells were dissociated with 0.5 mL Accutase for 3 min and seeded at a density of 10 000 cells cm^−2^, along with 0.5 µm Y27632 (ROCKi).

### Culture of XEn/EpiCs Within Thermoformed Microwells

The protocol had been adapted from Shankar et al., 2023.^[^ ^14b^ ^]^ The Epi/XEn‐induction medium containing advanced N2B27 medium was prepared as follows: 46.3% Advanced DMEM/F12 (Invitrogen), 46.3% Neurobasal (Invitrogen), 1% N2 supplement (Invitrogen), 2% B27 supplement (Invitrogen), 1% Glutamax, 1% Non‐Essential Amino Acids, 1.5% BSA (Sigma), 0.5% HEPES, 0.4% Sodium Pyruvate, 3 µm CHIR99021, 0.1 mm 2‐mercaptoethanol, 1mm 8Br‐cAMP, 25 ug mL^−1^ Fgf4, 1 ug mL^−1^ Heparin, 10 nm Retinoic acid, 1 µm Y27632, 5% FBS, and 1% Pen/Strep. 25 µL of the medium was added to all the wells and placed inside the incubator.

To remove dead cells, mESCs were washed twice with PBS, followed by treatment with 0.5 mL Accutase for 3 min. The cells were then centrifuged at 200 × *g* for 5 min, and the resulting pellet was resuspended in 7 mL of MEF medium composed of DMEM (high glucose, supplemented with Sodium pyruvate and Glutamax) and 15% FBS. For MEF depletion, the cell suspension was first seeded onto a non‐coated T75 flask and incubated for 20–30 min to allow MEFs to adhere to the surface. Without disturbing the adhered cells, the supernatant was carefully collected, centrifuged again, and the pellet was resuspended in 1 mL of advanced N2B27 medium. After cell counting, the suspension was adjusted to a concentration of 160 000 cells mL^−1^ using XEn‐ind medium, and 50 µL of this suspension was added to a tube containing 150 µL of XEn‐ind medium. Subsequently, 200 µL of the prepared cell suspension was dispensed into each well and incubated to allow cell settlement.

In all screening experiments, compounds were added to advanced N2B27 medium supplemented with 0.2% β‐Mercaptoethanol and 1% Penicillin/Streptomycin. The compound‐containing medium was added at 48 h and replaced with fresh medium at 72 h. For control conditions, 0.1% DMSO was added between 48 and 72 h. The culture was continued until 120 h, after which, the structures were processed for further analysis.

### Fixation and Staining

At 120 h, the structures were washed thrice with PBS and fixed for 30 min at room temperature using a fixation solution containing ice‐cold 2% paraformaldehyde (PFA) and 0.1% glutaraldehyde. Following fixation, the wells were washed three times with PBS and either processed for further staining or stored at 4 °C.

For staining, the structures were permeabilized with either 0.1% Triton X‐100 or 1% Tween‐20 for 30 min at room temperature. Subsequently, the wells were treated with Hoechst 33342 (1:300) and Phalloidin‐AF647 (1:300) in 0.1% Triton X‐100 and incubated for 30 min at room temperature. After incubation, the wells were washed three times with PBS and either stored at 4 °C or imaged using a microscope.

For caspase 3/7 staining, the fixed samples were first permeabilized with 0.1% Triton‐x 100 or 1% Tween‐20 for 30 min at RT, then blocked with blocking buffer (2% BSA, 5% serum of host 2nd AB species, 0.5% glycine, 0.1%, Triton‐X100, 0.2% Tween20) for 1 h and finally incubated with 1: 400 dilution of cleaved Caspase 3/7 primary antibody overnight at 4°. The samples were washed and incubated with secondary antibody conjugated with AF647 along with Hoechst and Phalloidin‐AF568 for 2 h at room temperature. The samples were washed 3× with 1 × PBS and stored.

### Imaging

Imaging of the structures was performed using an inverted Nikon Ti‐E live‐cell spinning disk confocal microscope, equipped with an environmental control system and a CrestOptics X‐Light V2 spinning disk unit. For all automated measurements, a 20× air objective (CLWD, O.D. = 2.10 mm, NA = 0.5) was employed. To capture the entire well, including all microwells, the ‘large image’ module was used to generate stitched montages. Manual image analysis, including yield and area measurements, was carried out using NIS software and ImageJ. All images were acquired using epi‐fluorescence imaging in a single focal plane, selected approximately at the plane where the cavity size was maximal. This imaging plane was chosen based on the assumption that most structures are spherical, and the cavity forms at the central plane of the structures (**Table** **3**).

**Table 3:** List of Antibodies and fluorescent stains.

Antibody/fluorescent stain	Company #catalog number	Dilution
Hoechst 33342 (nuclei stain)	Invitrogen #H3570	1:300
Phalloidin AF647 or AF568 (F‐Actin stain)	Invitrogen #10656353 (Alexa Fluor 647)	1:300
Rabbit anti‐cleaved Caspase 3/7	Cell signaling technology Asp175 #9661	1:400
Donkey anti‐rabbit AF647 (secondary antibody)	Invitrogen #10543623	1:300

### CellProfiler Pipeline Creation and Setup — Morphometrics (Measurement of Morphological Features)

The analysis of 3D structures was performed using CellProfiler (CP) v4.2.6 and CellProfiler Analyst (CPA) v3.0.4 (Broad Institute). Within each montage image of a well, individual structures were identified as distinct objects, and each object was quantified for gross feature measurements, including area, size, shape, texture, intensity, and intensity distribution. Zernike features were also included in the analysis. The quantification was performed using the modules ‘MeasureObjectArea,’ ‘MeasureObjectIntensity,’ ‘MeasureObjectIntensityDistribution,’ ‘MeasureObjectSizeShape,’ and ‘MeasureTexture.’ The resulting whole‐structure measurements were exported both into a database file for use with CPA and into a spreadsheet for further downstream analysis as used in Shankar et al., 2023.^[^ ^14b^ ^]^ All files and the pipeline can be shared upon request.

### Supervised Machine Learning

For supervised machine learning‐assisted training of the classification algorithm, individual structures (or objects) within each well were manually categorized into five distinct classes based on visual morphological features. These features included the presence of a polarized, rosette‐shaped epiblast (Epi), specification of Gata6‐positive cells, epithelialization of Gata6‐positive cells, and formation of a pro‐amniotic cavity in the center of the Epi. Using these visual criteria, objects were manually assigned to different classes to generate a training dataset. In the authors’ experiments, the training set size ranged from 25 to 30 structures per class. The training process involved evaluating and scoring the dataset using the ‘fast‐gentle boosting’ machine learning algorithm, which generated classification rules based on gross feature measurements obtained from CellProfiler. After supervised training, the performance of the classifier was assessed in terms of accuracy, precision, recall, and F1 score, yielding values of 85%, 84%, 76%, and 80%, respectively, for XEn/EpiC classifications, shown in detail in ref.[14b]

### Yield Quantification Using CellProfiler Analyst

The output data from the CellProfiler pipeline were imported into CellProfiler Analyst (CPA), where the classifier module was used to implement a supervised machine learning algorithm based on the ‘fast‐gentle boosting’ method. This algorithm applied the feature measurements obtained from CellProfiler to develop classification rules for each phenotype. To ensure consistency across different experimental rounds, additional structures were incrementally added to the training set to monitor and improve prediction accuracy. Once the training set was finalized, it was applied to score the entire dataset, encompassing all treatment and control conditions with replicates (≈288 wells per experiment, each well containing ≈160 structures; 160 × 288 = 46.080 structures were analyzed). This classification module provided quantitative output reflecting the yield of each structure class based on the extracted gross feature measurements.

In addition, the pipeline was trained similarly to classify the structures displaying a delaminated space between XEn and Epi compartments, which was used in identifying delaminated structures upon caffeine treatment.

### Quantification of the Area Using CellProfiler

The rules generated for each phenotypic class after the training with classifier module were imported back into the CP pipeline under the “Filter objects” module, and the objects falling into each class could be filtered out per image. The area of the phenotypic class ‘XEn/EpiCs’ was then measured using the ‘MeasureObjectArea’ module.

### Radial Intensity Distribution and Co‐Efficient Of Variance (RadialCV)

The automated image analysis pipeline could additionally measure the radial intensity distribution (Figure S7, Supporting Information) by segmenting each object (XEn/EpiCs or structures within microwells) into four radial bins. Fluorescence intensities of Caspase 3/7‐positive cells were quantified in each bin. As the 2D image of XEn/EpiCs corresponds to three distinct compartments—PAC, Epi, and XEn—these radial bins provided a spatial representation of each compartment. Hence, the radial intensity in the first bin would reflect the cell death in PAC, whereas the radial intensity in the third bin would reflect the cell death in Epi. The measure of cell death was quantified through the measurement of the co‐efficient of variance (RadialCV) for each bin (Figure S8, Supporting Information).

### ATP Assay

For the ATP measurement, the CellTiter Glo 3D luminescence kit was used where the reagent was added to the samples collected at 72 h after the exposure to treatment compounds, and at 120 h, after refreshing with fresh media. The luminescence measurement was calculated based on the instructions from the manufacturers. The amount of ATP measured was directly proportional to the number of live cells in the sample, giving information on the cytotoxic effects of different compounds.

### Statistical Analysis

All data obtained from CP consisting of yield ratios were pre‐processed by converting the different ratios of the phenotypes observed into percentage values per experiment using Microsoft Excel. All the area measurements obtained from the CP were directly imported into GraphPad Prism (v10.4.1) for further analysis. All data are presented as mean ± SD. All experiments were conducted in triplicates, with each treatment condition generating up to 169 structures per replicate. The sample size (*n*) for yield measurements was ≈ 160 structures. For area measurements, the XEn/EpiCs were filtered out based on their ratio within the whole population and were measured for all replicates (*n* = 3). The sample size for statistical analysis of area measurements could be in the range of 80–120 structures depending on the treatment condition. In the graphs, each dot represents one XEn/EpiC. All statistical hypothesis testing was done using Dunnett's test; ∗ represents *P* < = 0.05, ∗∗ represents *P* < 0.01, ∗∗∗ represents *P* < 0.001, and ∗∗∗∗ represents *P* < 0.0001 (one‐way ANOVA with Dunnet's post‐test).

## Conflict of Interest

S.Giselbrecht is one of the founders and shareholder of 300MICRONS.

## Supporting information



Supporting Information

## Data Availability

The data that support the findings of this study are available from the corresponding author upon reasonable request.
