# Supplementary material for: Morphometric‐Assisted Prediction of Developmental Toxicity Using Stem Cell‐Based Embryo Models in Microwells
Source: Adv Healthc Mater. 2025 May 9;14(15):2404847. doi: 10.1002/adhm.202404847 (PMC12147983; doi:10.1002/adhm.202404847)
Supplement: Supplementary file 1 — Supporting Information [file ADHM-14-0-s001.docx]

# **Supporting information**

## **Compound library:**

**Table S 1: Library of teratogens and non-teratogens and the concentrations used in this study**: concentrations in bold were used in secondary screen with 5 concentrations

|  | **No.** | **Compound** | **Type of molecule** | **FDA category** | **Concentrations** |
| --- | --- | --- | --- | --- | --- |
| Non - teratogens | 1 | Ascorbic acid | Water-soluble vitamin C. | A | **700 μM**, 432.0918 μM - 100μM - 10μΜ, **1 μM** |
|  | 2 | Isoniazid | Antibiotic for treatment of Mycobacterium tuberculosis | C | **364.59 μM**, **100 μM**, 32.0842 μM - 18.9589μΜ -364.5936nM |
|  | 3 | Penicillin G | Penicillin G is a narrow spectrum natural antibiotic. | B | **20 mM**, 2mM, 1mM, **200 μM**, 63μΜ |
|  | 4 | Folic acid | A nutritional delivery form of folate. Folic acid and its derivatives are essential mediators of one-carbon metabolism within cells. | A | 9.0621μM - 2.2655μΜ - 147.2587nM |
|  | 5 | Thiamine | Water-soluble vitamin-B complex. Thiamine is naturally available in fruits, vegetables, poultry, eggs. | A | **10 mM**, 1mM, 100μΜ, 10μΜm, **1 μM** |
|  | 6 | Aspirin | Aspirin (Acetyl Salicylic Acid) is a cyclo-oxygenase inhibitor and an antiplatelet agent, widely used for the secondary preventions of heart attack and stroke. | C (1st and 2nd trimester), D (3rd trimester) | 27.7534 mM - 5.5507mM -2.7753mM |
|  | 7 | Caffeine | Widely ingested pharmacologically active substance. It belongs to the class of methylxanthines. It is a naturally occurring alkaloid found in coffee, cocoa and other plants. | N/A | 2mM - 1mM - 500 μΜ |
|  | 8 | Diphenhydramine hydrochloride (DPH) | H1 histamine receptor antagonist | B | 391.6117nM - 195.8058nM - 39.1612nM |
|  | 9 | Ibuprofen | Non-steroidal anti-inflammatory drug (NSAID) | B (1st and 2nd trimester), D (3rd trimester) | **400 μM**, 200μΜ, 120μΜ, 63μΜ, **10 μM** |
|  | 10 | Indapamide | Indapamide is a non-thiazide sulphonamide diuretic compound, cAMP modulation; | B | 399.087μΜ, 199.5435μΜ, **100 μM**, 27.3347μΜ, **1 μM** |
| Teratogens | 11 | Dexamethasone (Hexadecadrol, Prednisolone) | Potent synthetic member of the glucocorticoid class of steroid drugs, and an interleukin receptor modulator that has anti-inflammatory and immunosuppressant effects. | C | **198.75 μM**, 100μM, **10 μM**, 1μΜ - 10nM |
|  | 12 | 5-Fluorouracil | 5-Fluorouracil has been used to induce apoptosis in cells. It has been used as a chemosensitizing agent. | D | 768.7695 μM, 76.877 μM, **7.68 μM,** **76.877 nM**, 768.7695 nM |
|  | 13 | Busulfan | Busulfan is a cell cycle non-specific alkylating antineoplastic agent. Busulfan induces apoptosis. | D | **500 μM**, 255.79 μM, **100 μM**, 40.6 μM, 2.03 μM |
|  | 14 | Cytosine arabinoside | It is a chemotherapeutic medication used to treat acute myeloid leukemia. | C/D | 100nM - 10nM - 1nM |
|  | 15 | Hydroxyurea | Anti-neoplastic. It blocks the synthesis of deoxynucleotides, which inhibits DNA synthesis and induces synchronization or cell death in S-phase. | D | 100nM - 50nM - 10nM |
|  | 16 | (*trans*) Retinoic acid ;ATRA, Tretinoin, Vitamin A acid, all-trans-Retinoic acid | RA is a metabolite of Vitamin A that is required for growth and development. | C | **1 μM**, 100 nM, 33 nM, **10 nM**, 0.4 nM |
|  | 17 | Thalidomide | Thalidomide has been used to study its neuropathological effects in mouse models of Alzheimer′s disease (AD). High risk of birth defects in embryos and fetus. | X | **200 μM**, 100 μM, 11 μM, 0.4 μM, **100 nM** |
|  | 18 | Valproic acid | Valproic acid's primary use is as an anti-seizure medication, as well as in migraine, bipolar, mood, and anxiety disorders. | D | **10 mM**, 1mM, 333μΜ, **100 μM**, 4μΜ |
|  | 19 | Carbamazepine | Carbamazepine is considered as a safe antiepileptic drug. | D | 397.9022 μM, 198.9511 μM, 42.33 μM, **10 μM**, **1 μM** |
|  | 20 | Cyclophosphamide | Alkylating chemotherapeutic agent | D | 0.589 mM- 95.748 μM- 19.149 μM |
|  | 21 | Isotretinoin (13-cis retinoic acid) | Isotretinoin was developed to be used as a chemotherapy medication for the treatment of brain cancer, pancreatic cancer and more. | X | **1 mM**, 1.664 μM, 0.333 μM, 0.0333 μM, **1 μM** |
|  | 22 | Etretinate (Tegison, Ethyl etrinoate, Retinoid, Etretinato,Ro 10-9359) | Etretinate is an oral aromatic retinoid acid which is effective in psoriasis and other dermatological syndromes. | X | 84.63 μM- 28.21 μM- 8.463 μM |
|  | 23 | Bosentan | Bosentan is an endothelin (ET) receptor antagonist for the treatment of pulmonary artery hypertension. | X | 18 μM, 8 μM, 4 μM, **1 μM**, **1 nM** |
|  | 24 | Phenytoin | Phenytoin is a hydantoin derivative, a first-generation anti-convulsant drug | D | 20μΜ - 5μΜ - 1μΜ |
|  | 25 | Propafenone hydrochloride | Beta adrenergic antagonist. Calcitonin, histamine, serotonin,estrogen R inhibitor. | C | 8.27μΜ, 1μΜ, 0.1μΜ, **10 nM**, **1 nM** |
|  | 26 | Trichlorfon | Antihelminthic (pro)drug, Cholinesterase inhibitor. Potent insecticide. In higher concentration toxic for the development of mouse embryos ^[40]^ | N/A | 466.128 μM- 155.376 μM- 19.422 μM |
|  | 27 | Ampyrone | Analgesic drug; Anti inflammatory; 4-Aminoantipyrine reduces toxic and genotoxic effects of doxorubicin, cisplatin, and cyclophosphamide in male mice ^[41]^ | N/A | 100 μM - 5μΜ - 0,2 μΜ |
|  | 28 | 2,4,6-triiodophenol (TIP) | Halophenolic disinfection byproducts, has been widely detected in water bodies, even in drinking water. | N/A | M - 10 μM - 5 μΜ |

**Supplementary figures**


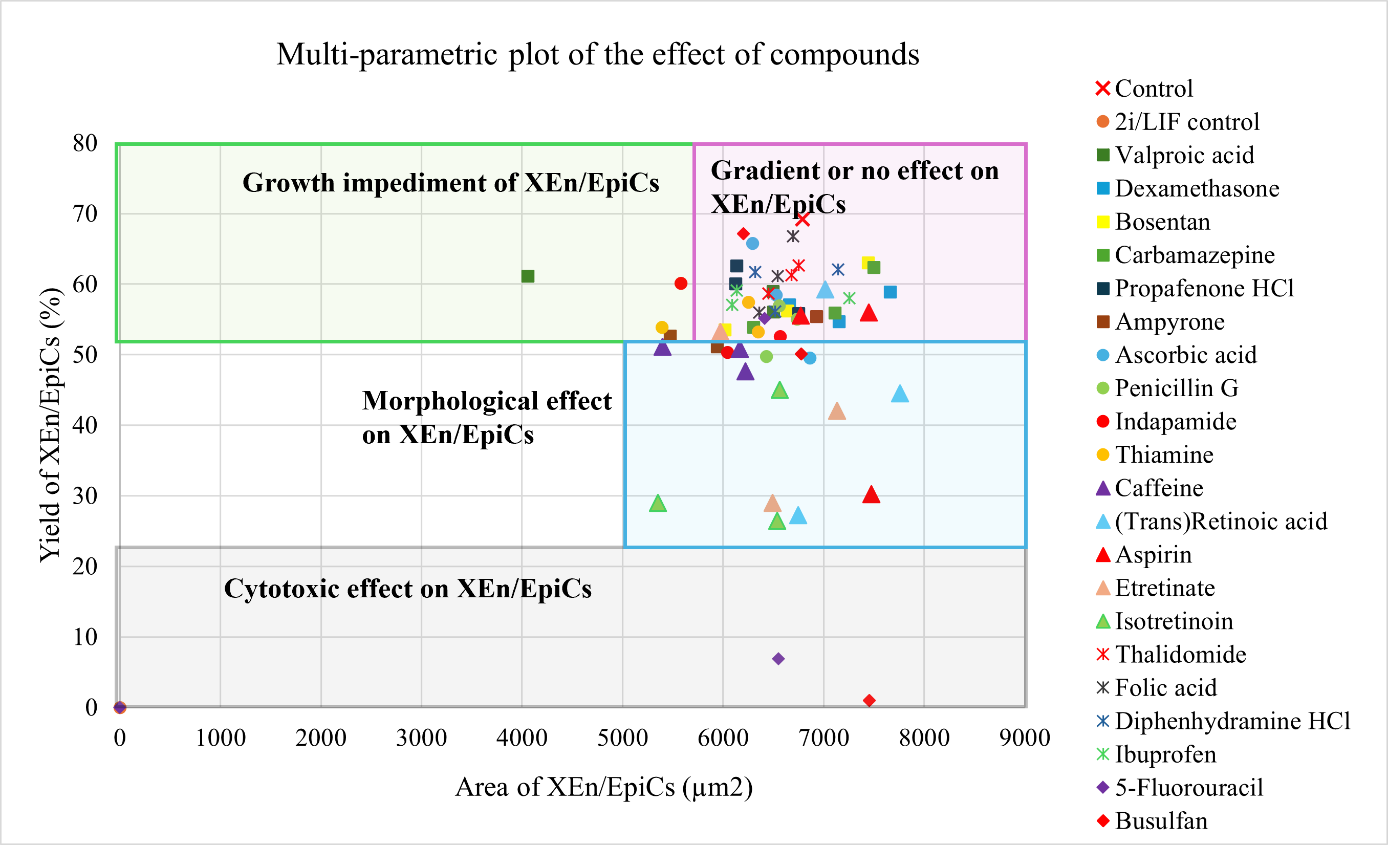


**Figure S 1: Multi-parametric phase analysis plot of all compounds grouped into the effects they showed.** Compounds inducing a gradient (dose-dependent) effect and those that did not show a significant effect on XEn/EpiCs development are grouped in the topmost right part of the plot; compounds that led to a morphological effect on XEn/EpiCs development are grouped in the middle right; and compounds that induced a cytotoxic effect grouped in the bottom of the graph. Some of the compounds induced a growth impediment, observed through a reduction in the overall area of XEn/EpiCs, grouped at the topmost left of the graph. The plot was generated using Microsoft Excel, by plotting a multi-variable scatter plot with average area on the X-axis and average yield on the Y-axis. Each point represents one compound at a specific dose. This graph was plotted based on the three-dose screening to classify the effects observed.


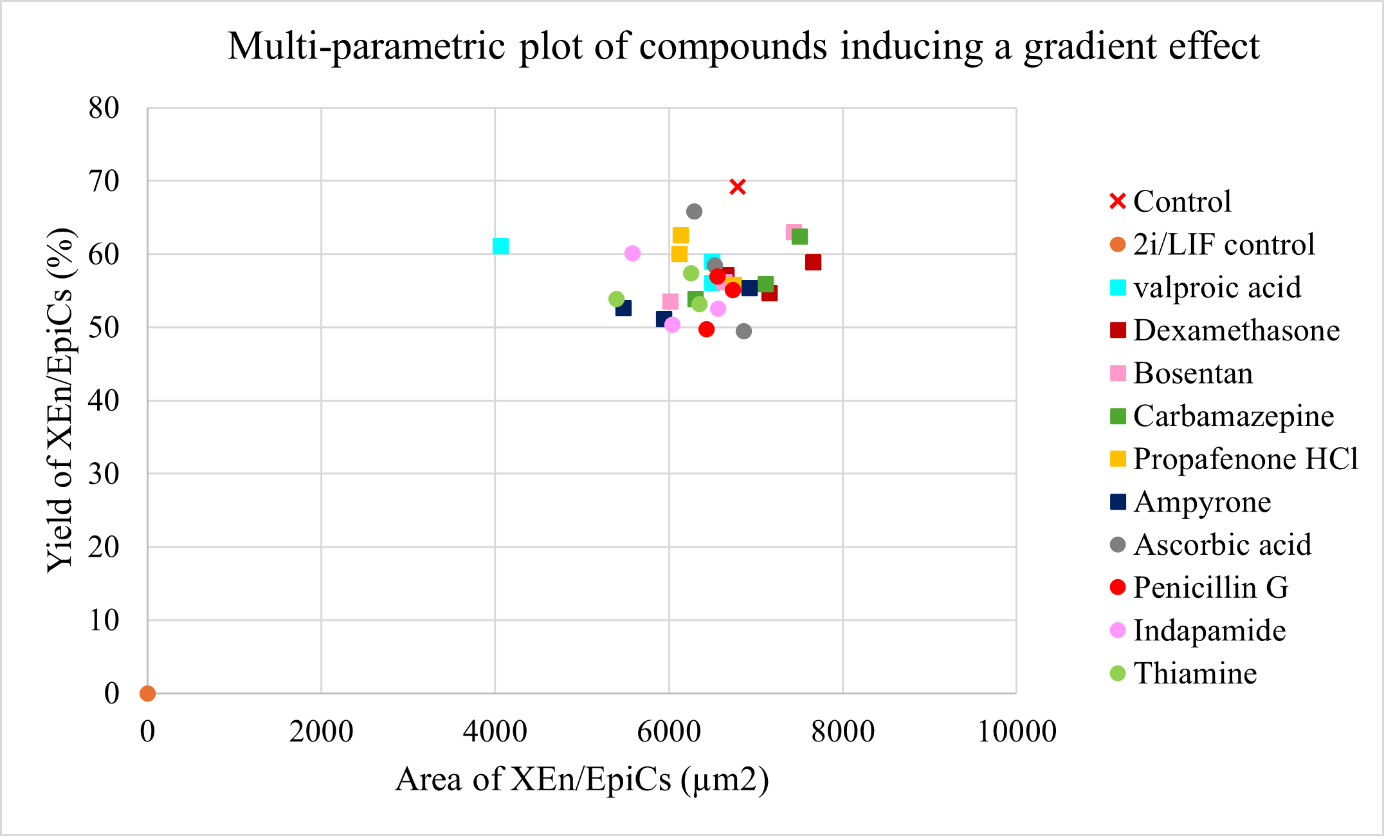


**Figure S 2: Multi-parametric phase analysis plot of the compounds inducing a gradient effect on the yield of XEn/EpiCs.** The squares represent teratogenic compounds, and the circles represent non-teratogenic compounds. The plot was generated using Microsoft Excel, by plotting a multi-variable scatter plot with average area on the X-axis and average yield on the Y-axis. Each point represents one compound at a specific dose. This graph was plotted based on the three-dose screening to classify the effects observed.


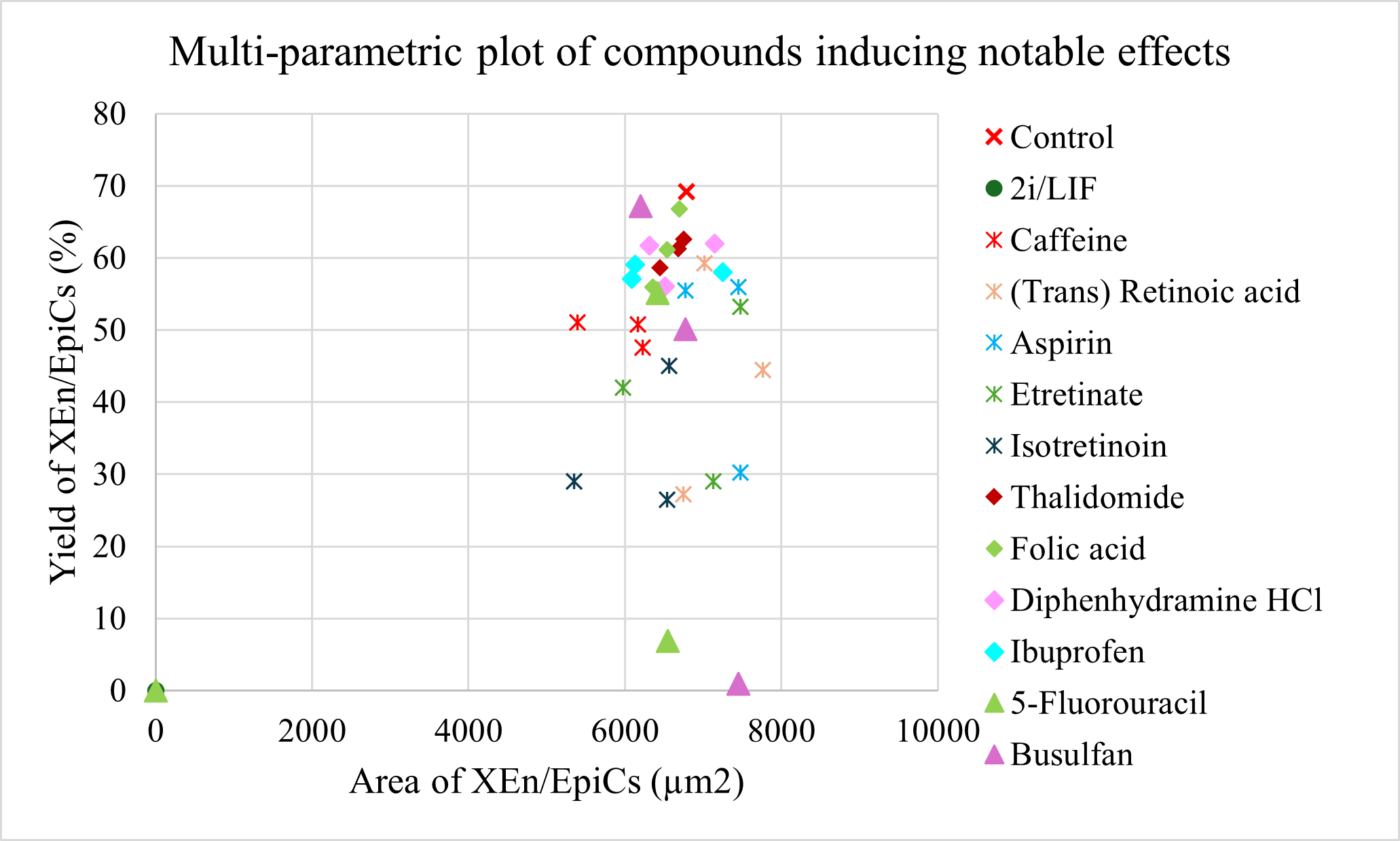


**Figure S 3: Multi-parametric phase analysis plot of the compounds inducing other notable effects on the yield of XEn/EpiCs.** The x’s represent compounds affecting the developmental progression of XEn/EpiCs, diamonds represent the compounds inducing no significant effect on the yield of XEn/EpiCs, and the triangles represent compounds causing a cytotoxic and morphotoxic effect on XEn/EpiCs. The plot was generated using Microsoft Excel, by plotting a multi-variable scatter plot with average area on the X-axis and average yield on the Y-axis. Each point represents one compound at a specific dose. This graph was plotted based on the three-dose screening to classify the effects observed.

**
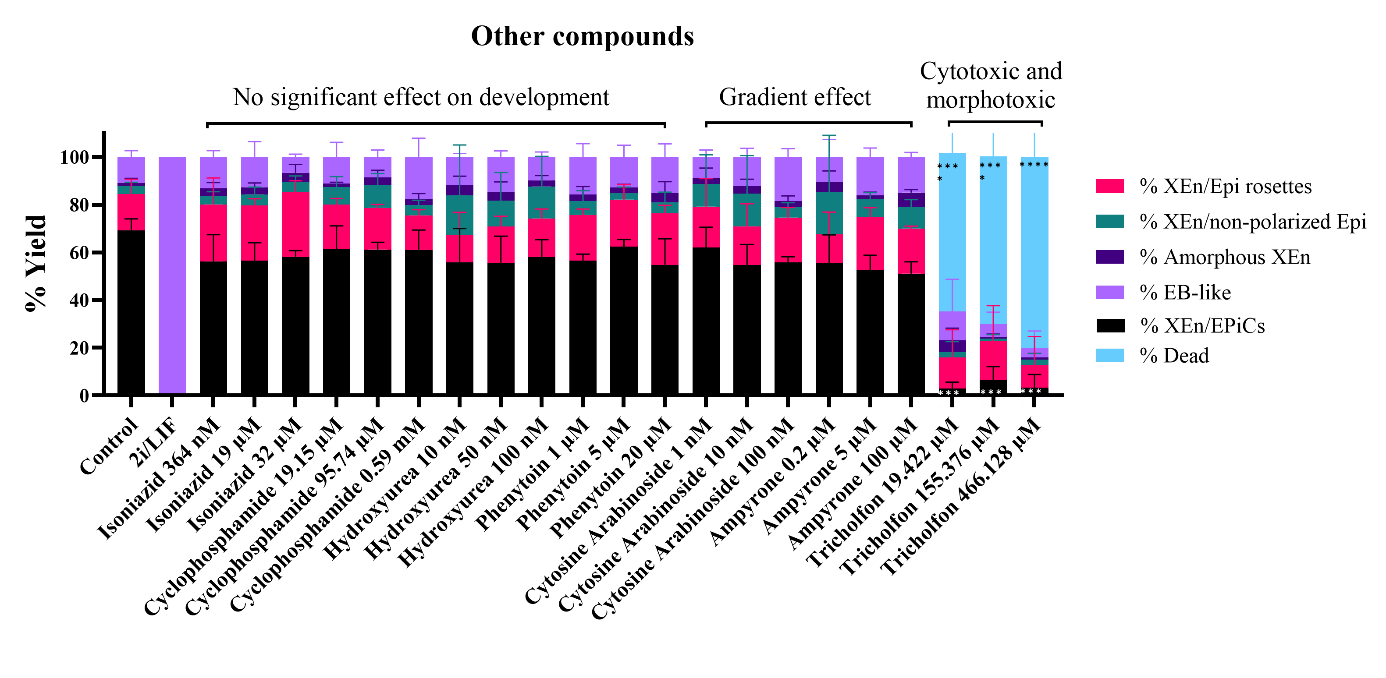
**

**Figure S 4: Effects of (remaining) compounds on the development of XEn/EpiCs:** Yield % of the five phenotypes, color-coded, are shown in the graphs. Data are mean ± s.d. obtained from n=3 wells, with each well containing ∼ 165 structures. Scale bar: 100 µm; All statistical hypothesis testing was done using Dunnett’s test; ∗ represents P <= ​0.05, ∗∗ represents P < ​0.01, ∗∗∗ represents P < ​0.001, ∗∗∗∗ represents P<0.0001 (One-way ANOVA with Dunnett’s post-test).


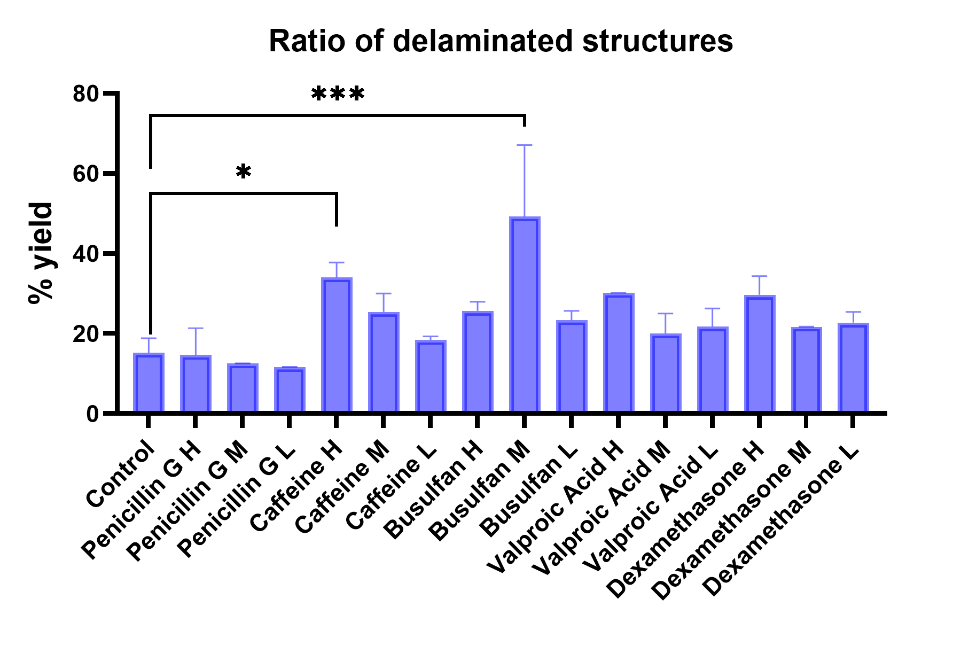


**Figure S 5: Percentage of delaminated structures in treatment conditions compared to the control**


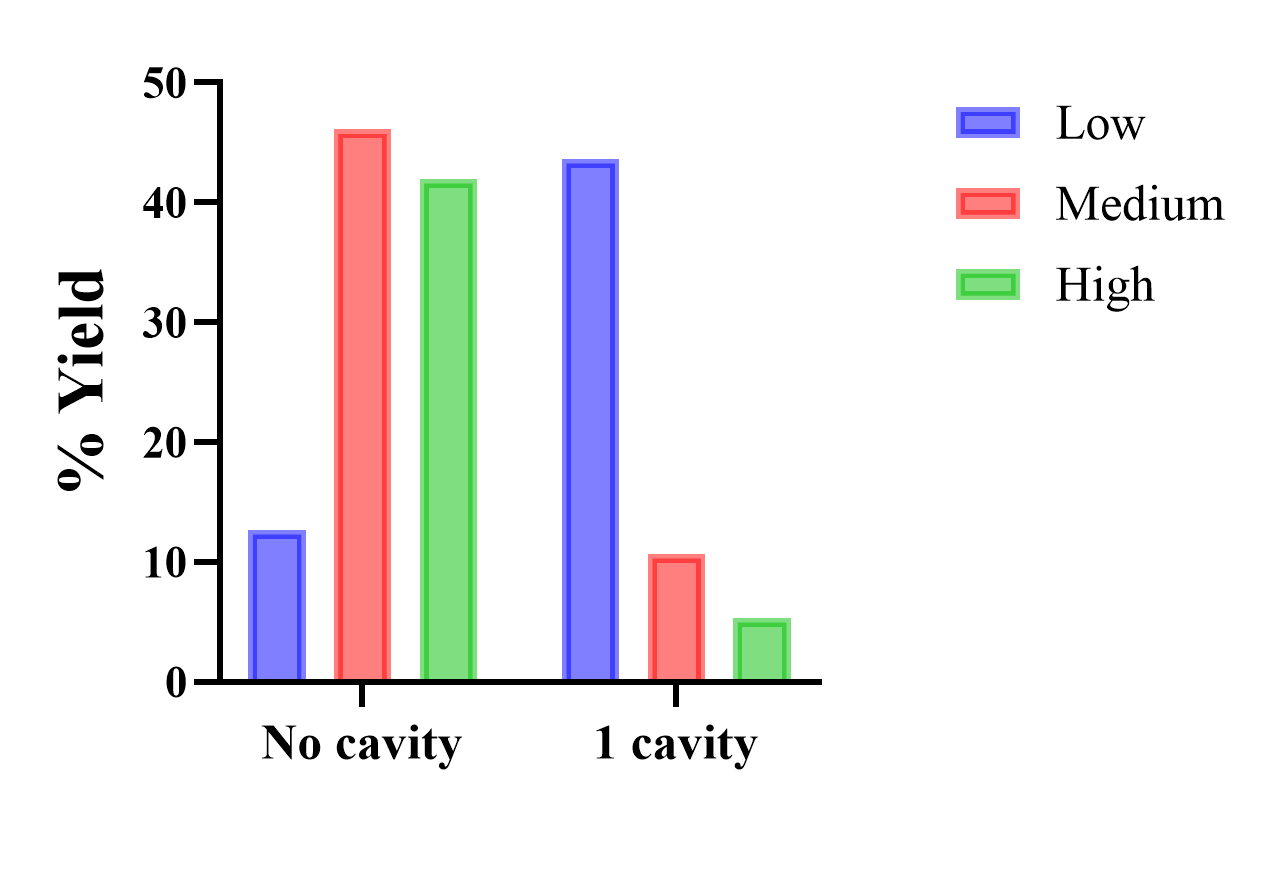


**Figure S 6: % Yield of single and no cavities in the XEn/EpiCs exposed to retinoic acid**


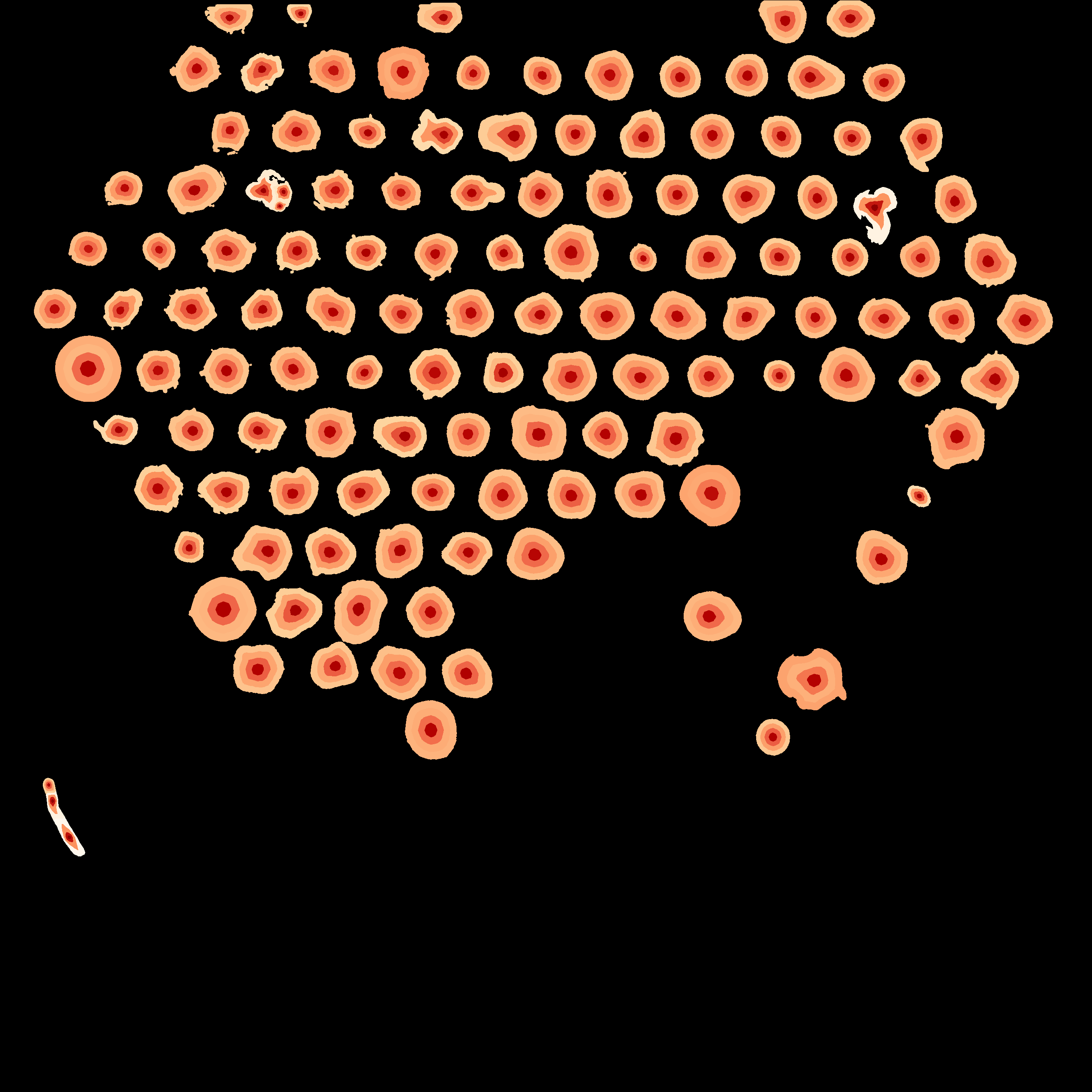


**Figure S 7: Quantification of fluorescence intensity distribution of Caspase 3/7 signal in control XEn/EpiCs**


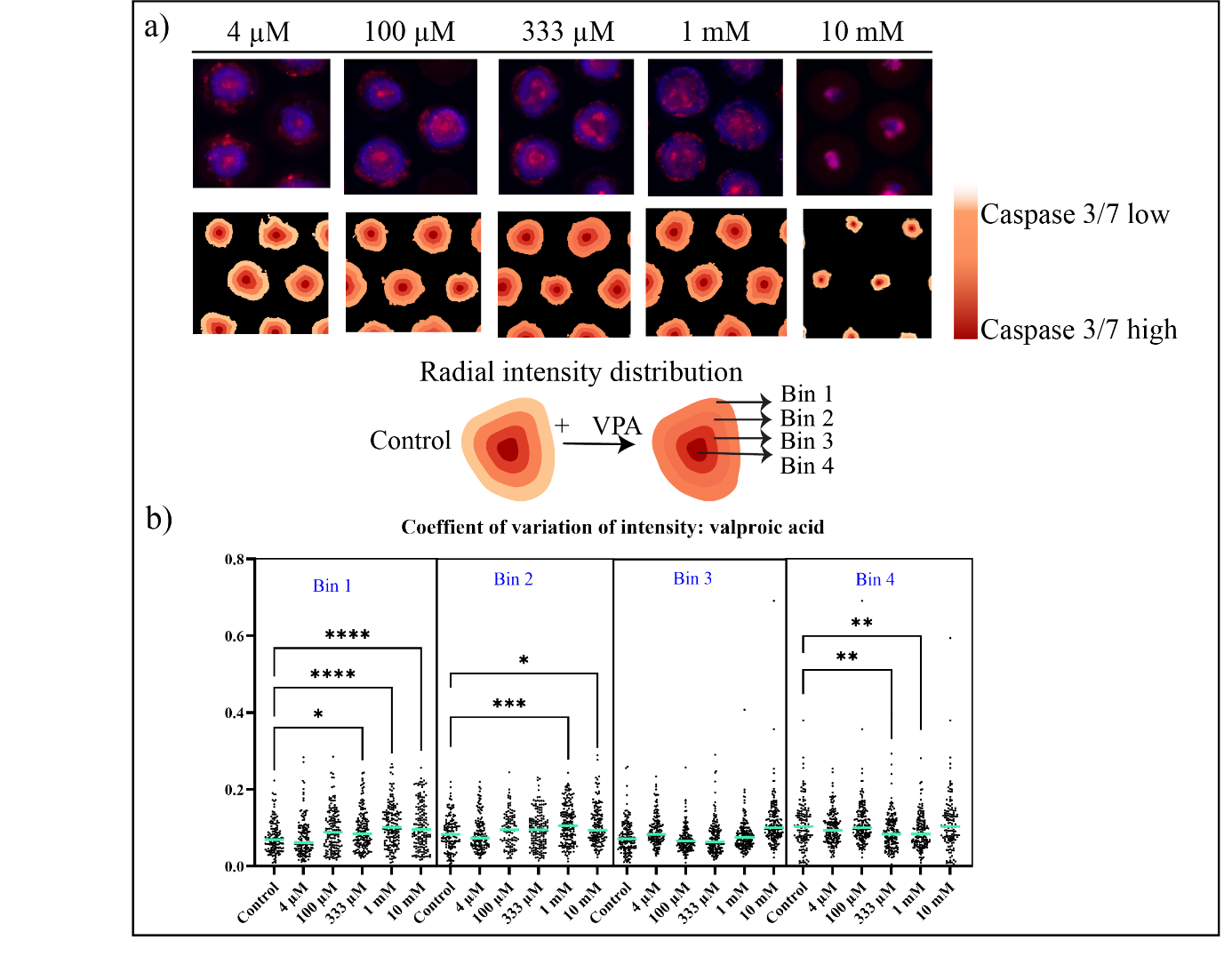


**Figure S 8: Radial intensity distribution of Caspase 3/7 quantified using CellProfiler**: a) (top) Caspase 3/7 antibody staining on 120h XEn/EpiCs after valproic acid (VPA) exposure. (bottom) Measurement of radial intensity distribution of caspase 3/7 staining using CellProfiler pipeline. The cartoon shows the division of one object (XEn/EpiCs) into 4 radial bins and the increase in red signal upon VPA exposure. B) Measurement of radial coefficient of variation of intensity between the four bins for the five doses of VPA. All statistical hypothesis testing was done using Dunnett's test; ∗ represents P< = 0.05, ∗∗ represents P < 0.01, ∗∗∗ represents P < 0.001, ∗∗∗∗ represents P < 0.0001 (One-way ANOVA with Dunnet's post-test).


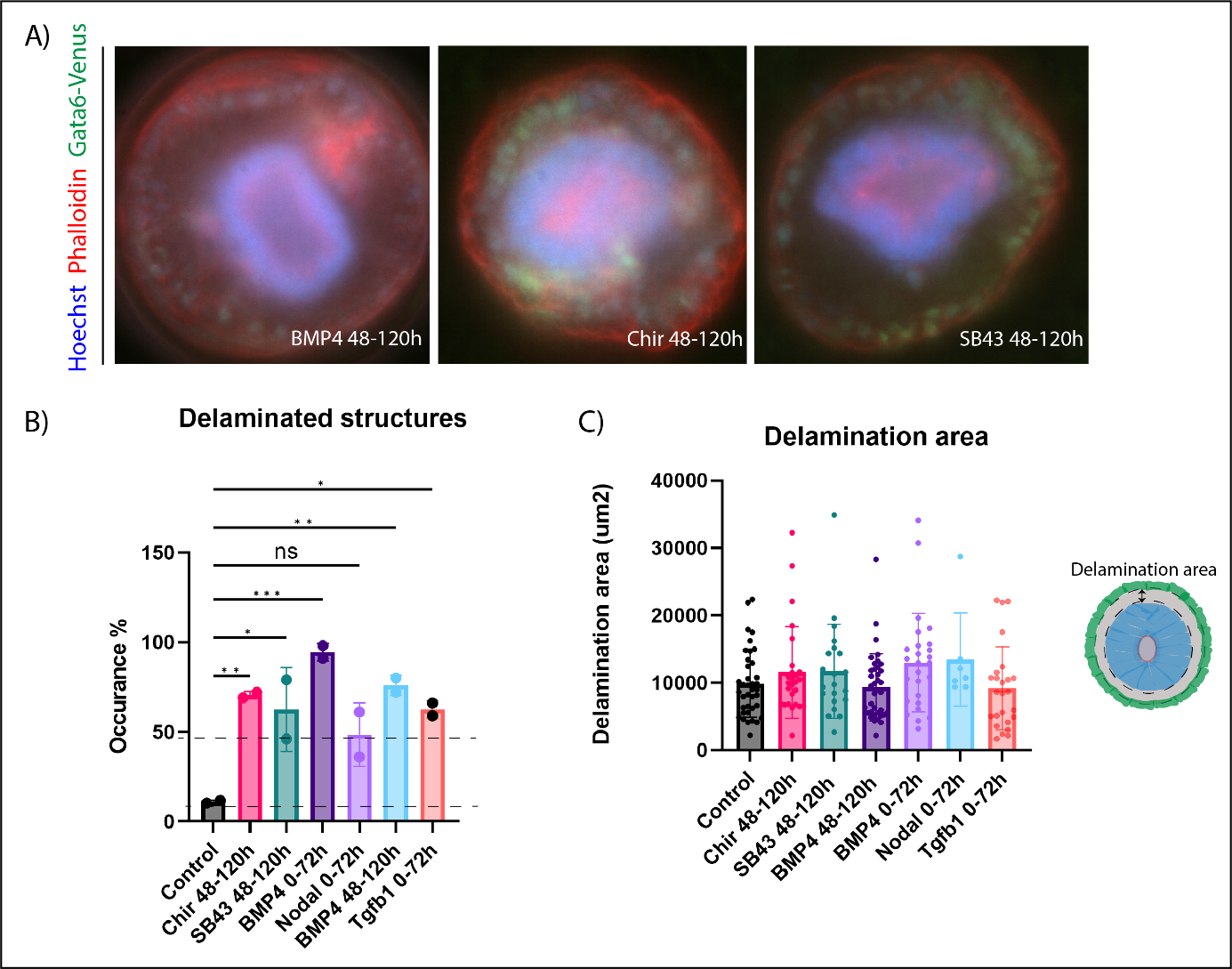


**Figure S 9: Delamination of XEn layer from Epi upon treatment with Wnt, BMP and Nodal pathway modulators:** A) XEn/EpiCs stained with Hoechst (nuclei) and Phalloidin (F-Actin) displaying delaminated XEn upon exposure to BMP4 (BMP activator), Chir99021 (Wnt activator), and SB43 (Nodal inhibitor). B) % occurrence of delaminated structures in different treatment conditions compared to control. C) Delamination area in XEn/EpiCs from different treatment conditions. All statistical hypothesis testing was done using Dunnett's test; ∗ represents P< = 0.05, ∗∗ represents P < 0.01, ∗∗∗ represents P < 0.001, ∗∗∗∗ represents P < 0.0001 (One-way ANOVA with Dunnet's post-test).
